# Nitidine chloride inhibits the progression of hepatocellular carcinoma by suppressing IGF2BP3 and modulates metabolic pathways in an m^6^A-dependent manner

**DOI:** 10.1186/s10020-025-01095-8

**Published:** 2025-02-05

**Authors:** Dan-dan Xiong, Zhen-dong Chen, Jian-di Li, Yu-long Deng, Rong-quan He, Zhi-guang Huang, San-qi An, Yi-wu Dang, Gang Chen

**Affiliations:** 1 Department of Pathology, The First Affiliated Hospital of Guangxi Medical University, Guangxi Zhuang Autonomous Region, Shuangyong RD 6, Nanning, 530021 P.R. China; 2 Guangxi Key Laboratory of Enhanced Recovery after Surgery for Gastrointestinal Cancer, The First Affiliated Hospital of Guangxi Medical University, Guangxi Zhuang Autonomous Region, Shuangyong RD 6, Nanning, 530021 P.R. China; 3 School of Basic Medical Sciences, Guangxi Medical University, Guangxi Zhuang Autonomous Region, Shuangyong RD 22, Nanning, 530021 P.R. China; 4 Department of Medical Oncology, The First Affiliated Hospital of Guangxi Medical University, Guangxi Zhuang Autonomous Region, Shuangyong RD 6, Nanning, 530021 P.R. China

**Keywords:** Nitidine chloride, Hepatocellular carcinoma, IGF2BP3, m^6^A, Metabolism

## Abstract

**Background:**

Hepatocellular carcinoma (HCC) stands as a major health concern due to its significant morbidity and mortality. Among potential botanical therapeutics, nitidine chloride (NC) has garnered attention for its potential anti-HCC properties. However, the underlying mechanisms, especially the possible involvement of the m^6^A pathway, remain to be elucidated.

**Methods:**

HCC cell and zebrafish xenograft models were utilized to validate the anti-HCC effects of NC. RNA-seq and MeRIP-seq analyses were performed to explore the potential targets and mechanisms of NC against HCC. The target effect of NC on IGF2BP3 was verified through RT-qPCR, WB, molecular docking, molecular dynamics (MD) simulation, surface plasmon resonance (SPR), and CCK8 off-target assays. Downstream target genes were confirmed using RNA stability assays.

**Results:**

In this study, utilizing HCC cell and zebrafish xenograft models, we validated NC’s ability to inhibit the growth, metastasis, and angiogenesis of HCC. Subsequently, employing RNA sequencing, RT-qPCR, WB, molecular docking, MD simulation, SPR, and CCK8 off-target assays, we pinpointed IGF2BP3 as a direct target of NC. IGF2BP3 is highly expressed in HCC, and IGF2BP3 knockdown significantly inhibited the proliferation, migration and invasion of HCC cells. Further MeRIP-seq and RIP-seq revealed 197 genes interacting with IGF2BP3, downregulated at mRNA and m^6^A levels after NC treatment, primarily associated with multiple metabolism-related pathways. Through intersection analysis, we pinpointed 30 potential metabolic target genes regulated by NC through IGF2BP3. Based on the expression of these genes, the metabolic scores for each HCC patient were calculated. Our findings suggest that patients with high metabolic scores have poorer prognoses, and the metabolic score serves as an independent prognostic factor. Finally, RNA stability experiments confirmed CKB, RRM2, NME1, PKM, and UXS1 as specific metabolic target genes affected by NC/IGF2BP3, displaying reduced RNA half-life post IGF2BP3 downregulation.

**Conclusion:**

Our study suggest that NC may exert its anti-HCC effects by downregulating IGF2BP3, inhibiting the m^6^A modification levels of metabolic-related genes, thereby reducing their stability and expression. Such insights provide a new direction in the study of NC’s anti-HCC mechanisms and offer novel perspectives for the treatment of HCC patients, focusing on both metabolic levels and m^6^A modification levels.

**Supplementary Information:**

The online version contains supplementary material available at 10.1186/s10020-025-01095-8.

## Introduction

Hepatocellular carcinoma (HCC), the main type of liver cancer, is one of the most common carcinomas with high morbidity and mortality globally, with over 90 thousand new diagnoses annually (Sung et al. 2021). Its prognosis remains grim, with a five-year relative survival rate standing below 20% (Siegel et al. 2022). Although the therapeutic landscape for HCC is diverse, advanced stages primarily lean towards systemic treatments, encompassing tyrosinase inhibitors and immune checkpoint inhibitors (Yang and Heimbach 2020). Nevertheless, challenges like drug resistance and tumor heterogeneity hinder the optimal efficacy of these modalities. In light of these challenges, the exploration of botanical-derived predictive biomarkers and therapeutic strategies may offer renewed hope for enhancing the survival and prognosis of HCC patients.

Nitidine chloride (NC) is an active ingredient mainly extracted from the roots or stems of Zanthoxylum nitidum (Roxb.) DC, a plant used in traditional Chinese medicine (Lu et al. 2022). It has been identified to perform antitumor activity in several malignancies, including HCC (Cui et al. 2020). Previous studies have reported that NC induced apoptosis and inhibited cell growth in HCC cells by targeting the JAK1/STAT3 (Liao et al. 2013) and JNK/c-Jun (Chen et al. 2022) signaling pathways. Our previous studies have also demonstrated that NC can exert anti-HCC effects by mechanisms involving multiple networks of epigenetic regulation, such as cicRNA-miRNA-mRNA (Xiong et al. 2019), lncRNA-TF-mRNA (Gao et al. 2019b), and TF-miRNA-mRNA (Gao et al. 2019a). However, the molecular mechanisms underlying NC anti-HCC properties still require more in-depth elucidation and research.

N6-methyladenosine (m^6^A) modification is the most prevalent form of RNA modification in the epitranscriptome. It plays a crucial role in regulating gene expression by impacting several facets of mRNA metabolism, including RNA alternative splicing, nuclear export, mRNA degradation, mRNA stability, and translation (He and He 2021). Dysregulation of m^6^A modification can lead to the onset and progression of various diseases, including pulmonary fibrosis (Lu et al. 2023), cervical cancer (Mao et al. 2023) and HCC (Liu et al. 2023a; Yang et al. 2022). Some m^6^A regulators in HCC have been reported. For example, the m^6^A methyltransferase, METTL3, can act as an m^6^A writer to promote HCC progression and drug resistance by enhancing m^6^A modifications on oncogenic transcripts (Pan et al. 2021). FTO, an m^6^A demethylase, is known as an m^6^A eraser and was found to promote HCC growth, metastasis and stemness by reducing the m^6^A abundance of GPNMB, stabilizing it from degradation by YTHDF2 (Chen et al. 2025). Studies have also reported that traditional Chinese medicine can influence disease progression by modulating m^6^A modifications (Dang et al. 2020). However, there is currently a lack of research on whether traditional Chinese medicine can effectively inhibit the progression of HCC through m^6^A modulation. Nevertheless, there is yet to be any report on whether NC can inhibit HCC progression by modulating m^6^A modifications.

In this study, leveraging a combination of in vitro and in vivo experiments, RNA sequencing (RNA-seq) and methylated RNA immunoprecipitation sequencing (MeRIP-seq), we uncovered for the first time that NC may exert its anti-HCC properties by inhibiting the m^6^A reader protein IGF2BP3, thereby regulating the m^6^A modification of key genes and influencing their expression. IGF2BP3, belonging to the insulin-like growth factor 2 mRNA-binding protein family, stands out as a significant m^6^A reader. This family enhances the stability and translation of target mRNAs, emphasizing m^6^A modifications, playing a pivotal role in post-transcriptional gene regulation and the wider landscape of cancer biology (Huang et al. 2018). Distinctively, aberrant IGF2BP3 expression has been pinpointed across diverse malignancies, including leukemia, colorectal cancer, and notably HCC. Its influence spans pivotal cancer biology spheres, such as cellular growth, migration dynamics, metabolic shifts, resistance to treatments, and immune interplay (Liu et al. 2023b; Zhu et al. 2023). Given these insights, IGF2BP3 emerges as a promising avenue for biomarker discovery and offers potential as a botanical therapeutic target in oncology.

**Fig. 1:**
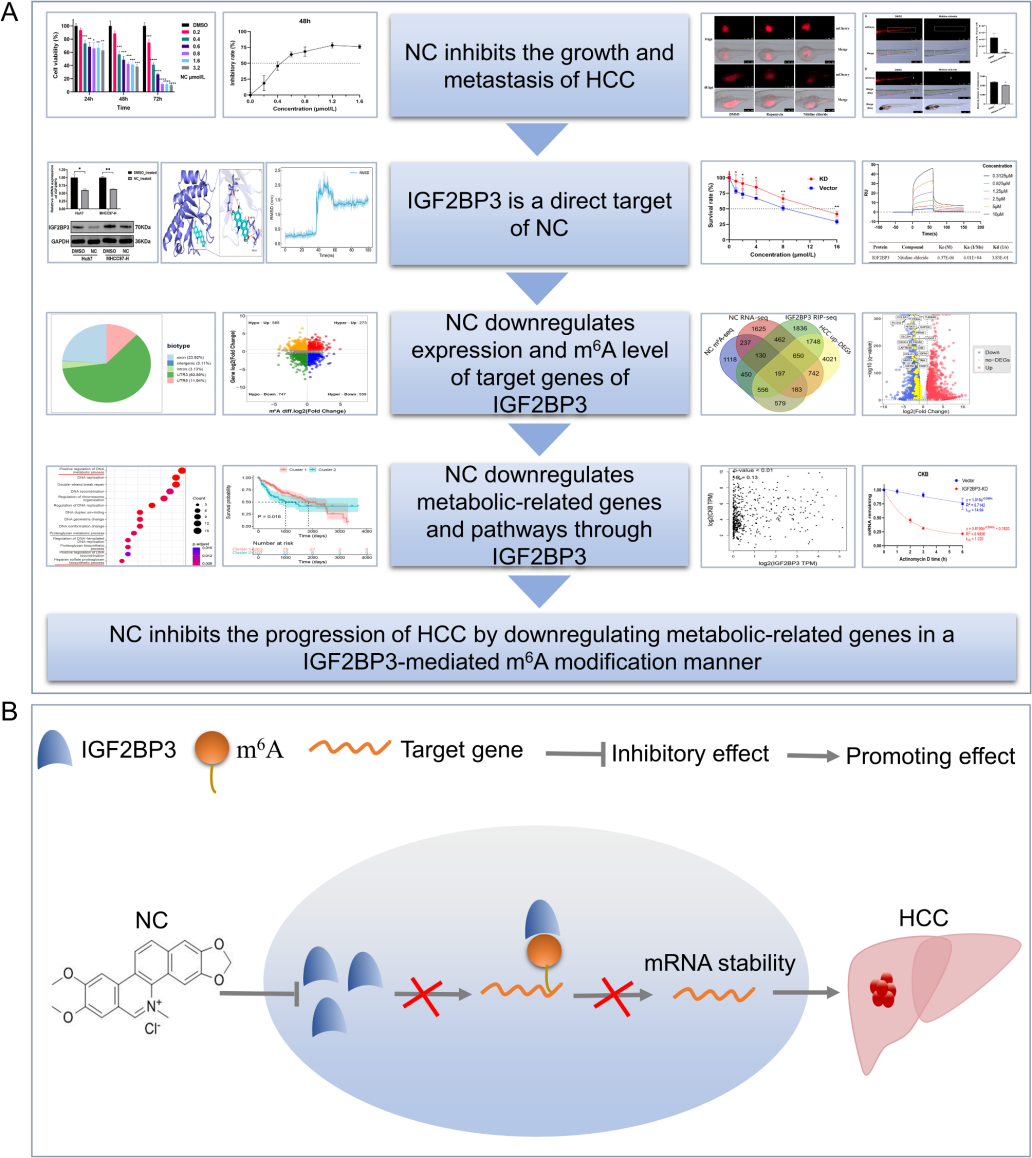
Overall design and hypotheses of this study. **A** Overall design of this study; **B** Hypotheses of this study. NC, nitidine chloride; HCC, hepatocellular carcinoma

In our pursuit to comprehend the molecular mechanisms by which NC impedes HCC progression post-downregulation of IGF2BP3, we amalgamated IGF2BP3-centric RNA immunoprecipitation sequencing (RIP-seq), real-time quantitative polymerase chain reaction (RT-qPCR), and other bioinformatic methods, and found that metabolic pathways and associated genes may play a significant role in this context. Altogether, our study reveals that NC may exert anti-HCC effects through the IGF2BP3/m^6^A/metabolic gene regulatory axis, which contributes to further research and clinical applications of NC as an anti-HCC treatment. The overall design and hypotheses of the current study are shown in Fig. 1.

## Materials and methods

### Cell culture

The Huh7 cell line was purchased from the Chinese Academy of Sciences Cell Bank (Shanghai, China), and the MHCC97-H cell line was purchased from Cellcook Biotech Co., Ltd. (Guangzhou, China). All cells were cultured at 37℃ using Dulbecco’s modified Eagle’s medium (DMEM, Gibco, USA) supplemented with 10% fetal bovine serum (FBS, Biological Industries, Israel) and 1% penicillin/streptomycin (Beijing Solarbio Science & Technology Co.,Ltd., China) in an incubator with 5% CO2.

### Drug preparation

NC (96% purity) was obtained from Chengdu Herbpurify Co., LTD. (Chengdu, China). The NC was dissolved in dimethyl sulfoxide (DMSO), and then further diluted to the working concentration with medium.

### In vivo studies in zebrafish

Zebrafish transplantation tumor models were established by microinjecting Huh7 and MHCC97-H cells, labeled with CM-DiI fluorescent dye, into the yolk sacs (for tumor growth studies) or yolk interstitials (for tumor angiogenesis and metastasis studies) of wild-type AB, Tg (kdrl: EGFP) and Tg (fli1a: EGFP) zebrafish embryos at 2 days post fertilization by microinjection, with 400–900 cells per embryo. All zebrafish were raised at 28℃ with specialized fish-culturing water. At 24 h after fertilization, 1-phenyl-2-thiourea (PTU) was added to inhibit melanogenesis. One day after tumor transplantation, drugs were administered via yolk sac injection at 24-hour intervals. Subsequently, the transplanted tumors before and after drug administration were photographed using in vivo fluorescence microscopy. The Fiji software was then utilized to analyze the effects of NC on the growth, angiogenesis, and metastasis of zebrafish transplanted tumors. After the experiment, zebrafish were anesthetized and humanely euthanized using tricaine methanesulfonate. All zebrafish experiments were approved by the Animal Research Committee of the First Affiliated Hospital of Guangxi Medical University and conducted in accordance with the National Research Council’s Guide for the Care and Use of Laboratory Animals.

### Lentiviral transfection

Lentiviral vectors for IGF2BP3 knockdown and control vectors were purchased from Gikai (Shanghai, China). Cells were transfected with lentivirus at MOI = 20 and cell screening was performed in medium containing 2.5 µg/mL puromycin (Servicebio, China) to obtain IGF2BP3 knockdown cell lines with stable expression.

### RT-qPCR

Total RNA was extracted from cells using the AxyPrep Total RNA Small Volume Preparation Kit (Axygen, USA). RNA concentration was determined using a NanoDrop 2000. Reverse transcription was performed using the HiScript III RT SuperMix (+ gDNA wiper) kit (Vazyme, Nanjing, China) according to the instructions. RT-qPCR was performed using the 2X Universal SYBR Green Fast qPCR Mix (ABclonal, Wuhan, China) on the LightCycler96 system. GAPDH and B2M were employed for result normalization, and quantification of relative mRNA expression was achieved using the 2–∆∆Ct method. The primer sequences used are provided in Table S1.

### Western blotting (WB)

Cell protein extraction was carried out using Cell Lysis Buffer for Western and IP (Beyotime, China). Following protein quantification and denaturation, an equal amount of protein samples was loaded onto pre-prepared 10% PAGE gels, subjected to electrophoresis, and subsequently transferred onto 0.22 μm PVDF membranes (Biosharp, China). The membrane was then blocked with 5% skimmed milk for 1.5 h and incubated overnight at 4 °C with the relevant primary antibody. Following three TBST washes, secondary antibodies were introduced and incubated for 1 h at room temperature. Chemiluminescence-based imaging was conducted using an imaging system (CLiNX, China). The antibodies used are as follows: IGF2BP3 Rabbit mAb (ab179807, Abcam), GAPDH Rabbit pAb (AC001, ABclonal), and HRP Goat Anti-Rabbit IgG (H + L) (AS014, ABclonal).

### Cell counting kit-8 (CCK8) assay

Cell viability was assessed using the Cell Counting Kit-8 reagent (Vazyme, China). HCC cells were seeded in 96-well plates at a density of 3000 cells per well. Depending on the experimental design, NC treatment was administered or omitted. At time points of 0 h, 24 h, 48 h, 72 h, and 96 h, 10 µL of CCK8 reagent (diluted in 100 µL of medium per well) was added and incubated with the cells at 37 °C in an incubator with 5% CO2 for 1 h. The absorbance at 450 nm of each well was measured using a microplate reader (Bio-Rad, USA).

### Scratch assay

Cells were pre-seeded into 6-well plates. When reaching 90% confluence, scratches were manually created. The medium was refreshed every 24 h with DMEM containing 1% FBS, and microscopic images were captured. Each cell type was plated in triplicate, and at least six fields were randomly captured per well. The area of wound closure was quantified using Image J software.

### Transwell migration and invasion assays

Cells were resuspended in serum-free medium and counted. 7 × 10^4^ cells suspended in 100 µL of serum-free medium were seeded into the upper compartment of the transwell chamber for migration assays. For invasion assays, 1 × 10^5^ cells/100 µL were seeded into the upper chamber pre-coated with 60 µL of Matrigel diluted 1:8 in serum-free medium. The lower chamber was filled with 500 µL of medium containing 10% FBS. After 24 h of incubation, the transwell chambers were removed, and the inner walls were gently wiped. The transwell chambers were then washed with PBS, fixed with 4% paraformaldehyde, and stained with 0.3% crystal violet. Finally, four random fields were captured per well under an inverted microscope.

### RNA stability assay

Cells were seeded into six-well plates at an appropriate density, and once the cell confluence reached 70–80%, 5 µg/mL actinomycin D (Biotopped, China) was added to treat the cells for 0 h, 1 h, 2 h, 3 h, 4–6 h, respectively. Subsequently, total RNA extraction and RT-qPCR were performed. After obtaining the Ct values, the relative abundance of mRNA at each time point relative to 0 h were calculated and then plotted using GraphPad Prism. The mRNA decay rate was further determined through nonlinear regression curve fitting (single-phase decay model) (Ratnadiwakara and Änkö 2018).

### RNA-seq and MeRIP-seq

Huh7 cells were subjected to a 48-hour treatment with 4 µmol/L of NC. Both NC-treated and DMSO control groups were established, each comprising three replicates. After the treatment using TRIzol Reagent (Sangon, China), cell samples were submitted to the company for RNA-seq and MeRIP-seq. Differential expression analysis was performed for both RNA-seq and MeRIP-seq results on R 4.2.1 using the “DESeq2” package. The criteria used to identify differentially expressed genes (DEGs) were|log2 (fold change)| > 0.6 and *P* < 0.05. For differentially m^6^A-modified peaks, the criteria were|log2 (fold change)| > 0 and *P* < 0.05.

### Molecular docking

The PDB Fomat of IGF2BP3 (PDB ID, 6GQE) was downloaded from the PDB (http://www.rcsb.org/pdb/home/home.do) website. The 3D structure of NC was acquired from PubChem (https://pubchem.ncbi.nlm.nih.gov/). Structural preparations such as dehydrogenation and hydrogenation of the acquired protein 3D structures were performed using Pymol software. Blind docking was performed using the CB-DOCK2 online server, which performed cavity detection based on an artificial neural network and utilized Autodock Vina for docking (Liu et al. 2022). Upon completion of the docking, the one with the lowest binding energy was selected as the best conformation, and further analysis of intermolecular interactions and binding site evaluation was conducted to assess stability for subsequent dynamic validation. Interaction analysis between the ligand and receptor was carried out using the PLIP online server (https://plip-tool.biotec.tu-dresden.de/plip-web). The 3D conformations of the ligand-receptor complexes were visualized using Pymol software.

### Molecular dynamics (MD) simulation

All-atom MD simulations were performed using the ligand-protein complexes obtained from the docking results as the initial structures. Simulations were conducted using Gromacs 2023.3 software. Both the ligand and protein were described using the AMBER protein force field (Maier et al. 2015; Wang et al. 2004). The system was prepared with the addition of hydrogen atoms via the pdb2gmx module, followed by the creation of a truncated cubic TIP3P solvent box (Mark and Nilsson 2001) at a distance of 10 Å. Na⁺ or Cl⁻ was added to neutralize the system’s charge. The topology and parameter files for simulation were then generated.

The MD simulations, lasting 100 ns, were performed using Gromacs 2023.3. Before the simulation, energy minimization was conducted using the steepest descent method with the initial step size set to 0.01 nm and a maximum force tolerance of 1000 kJ/mol•nm. After energy minimization, the system underwent a 100 ps NVT (constant volume and temperature) simulation to gradually heat the system from 0 K to 310.15 K, allowing solvent molecules to distribute uniformly. This was followed by a 100 ps NPT (constant pressure and temperature) simulation using the Berendsen barostat to equilibrate the system’s pressure at 1 bar. During the MD simulation, all hydrogen bonds were constrained using the LINCS algorithm with a 2 fs integration step. Electrostatic interactions were calculated using the Particle-mesh Ewald (PME) method with a cutoff of 1.2 nm, while non-bonded interactions used a cutoff distance of 10 Å, updated every 10 steps. Post-simulation, the trajectory was processed for periodic boundary condition removal, followed by analyses such as root mean square deviation (RMSD), root mean square fluctuation (RMSF), radius of gyration (Rg), and hydrogen bond counts.

### Surface Plasmon Resonance (SPR)

Experiments were performed at 25 ℃ on a BIAcore T200 using CM5 sensor chips, and data were analyzed using BIAcore T200 Evaluation software (GE Healthcare) following the manufacturer’s instruction. In brief, a cell on the CM5 sensor chip was activated with a mixture of 200 µM 1-ethyl-3-(3-dimethylaminopropyl) carbodiimide (EDC, GE Healthcare) and 50 µM N-hydroxysuccinimide (NHS, GE Healthcare) at 10 µL/min for 420 s. The IGF2BP3 protein was diluted to 50 µg/mL with sodium acetate (pH 5.0) solution and immobilized on the surface of the cell at 10 µL/min for 420 s for two repetitive runs. The cell was then blocked with 1 M ethanolamine (10 µL/min for 420 s). A neighbouring aisle that served as a reference was similarly activated and blocked, except that protein-free sodium acetate solution was used for immobilization. Both of the aisles were then equilibrated with PBS. Nitidine chloride stock solution was diluted to a series of concentrations in PBS, and was flowed at 30 µL/min for 150 s in each run. At the end of each flow, cells were regenerated for 5 min with 10 mM glycine-HCl (pH 2.0) solution at 10 µL/min. Data from the sample cell were collected using BIAcore T200 Control software (v. 2.0, GE Healthcare), and were subtracted from those from the reference cell. Association and dissociation constants were obtained by global fitting of the data to a 1:1 Langmuir binding model using BIAcore T200 Evaluation software (v.2.0, GE Healthcare). Data were exported to Origin 7 software (v.7.0552, OriginLab) for generating the final figures.

### Expression analysis and prognostic value of IGF2BP3 in HCC

Diverse global datasets related to HCC and normal liver tissues were collected from Gene Expression Omnibus (GEO) (Barrett et al. 2012), ArrayExpress (Sarkans et al. 2021), The Cancer Genome Atlas (TCGA) (Tomczak et al. 2015) and Genotype-Tissue Expression (GTEx) (Consortium 2013) databases. The databases from the same platform were then merged, de-batched using the ComBat function from the “sva” package (Leek et al. 2012), and normalized by log2(x + 1). Then DEGs were assessed by calculating the standardized mean difference (SMD). When the SMD value was above 0 and the 95% confidence interval (CI) didn’t encompass zero, it was identified as an upregulated gene. Conversely, if SMD was less than 0 and the 95% CI didn’t contain zero, it was classified as a downregulated gene.

IGF2BP3 mRNA expression was analyzed by integrating the included datasets by calculating SMD as described previously, and forest plots were plotted using the “meta” package. Then the summary receiver operating characteristic (SROC) curve was further plotted to evaluate the ability of IGF2BP3 expression to discriminate between HCC and non-HCC tissues. The IHC images of IGF2BP3 expression in HCC and normal liver tissues were downloaded from the Human Protein Atlas database (Uhlén et al. 2015). The prognostic role of IGF2BP3 in HCC was assessed based on the Kaplan-Meier plotter database (Győrffy 2021). When the hazard ratio (HR) value was greater than 1, the corresponding factor was considered to be a risk factor for patient survival.

### Acquisition of IGF2BP3 related datasets

Genes capable of directly binding to IGF2BP3 were derived from GSE90639, a RIP-seq dataset of IGF2BP3. Genes differentially downregulated by IGF2BP3 knockdown were obtained from GSE90684, a dataset of RNA-seq performed on IGF2BP3 knockdown and control HepG2 cells, and analysis was conducted using GEO2R with screening criteria of |log2 (fold change)| > 0.7 and *P* < 0.05. 3463 metabolic-related genes derived from 114 metabolic pathways were identified by SR Rosario et al. (Rosario et al. 2018).

### Enrichment analysis

Gene Ontology (GO) and Kyoto Encyclopedia of Genes and Genomes (KEGG) enrichment analyses were conducted using the “clusterProfiler” and “org.Hs.eg.db” packages. Following the acquisition of enriched pathway information, the “ggplot2” package was employed to create bubble plots or histograms for presentation.

### Calculation of metabolic scores of HCC patients

Single-sample gene set enrichment analysis (ssGSEA) of 374 HCC samples from TCGA was conducted with the GSVA package in R, and the metabolic score for each HCC patient was calculated (Hänzelmann et al. 2013). Based on the metabolic scores, patients were categorized into high and low metabolic groups. Further investigations were performed to examine the correlation between metabolic scores/groups and IGF2BP3 expression, in addition to their relationship with various clinicopathologic parameters such as age, race, survival status, T stage, TNM stage, and histologic grade. Additionally, the prognostic significance of the metabolic scores was assessed using Kaplan-Meier plots and both univariate and multivariate Cox regression analyses.

### Statistical analysis

Statistical analysis and plotting were conducted utilizing GraphPad Prism 9 and R v.4.2.3. Student’s t-test or Mann–Whitney U-test was employed to assess differences between two groups of data. *P* values less than 0.05 were statistically significant.

## Results

### NC inhibited the proliferation of HCC cells in vitro

**Fig. 2:**
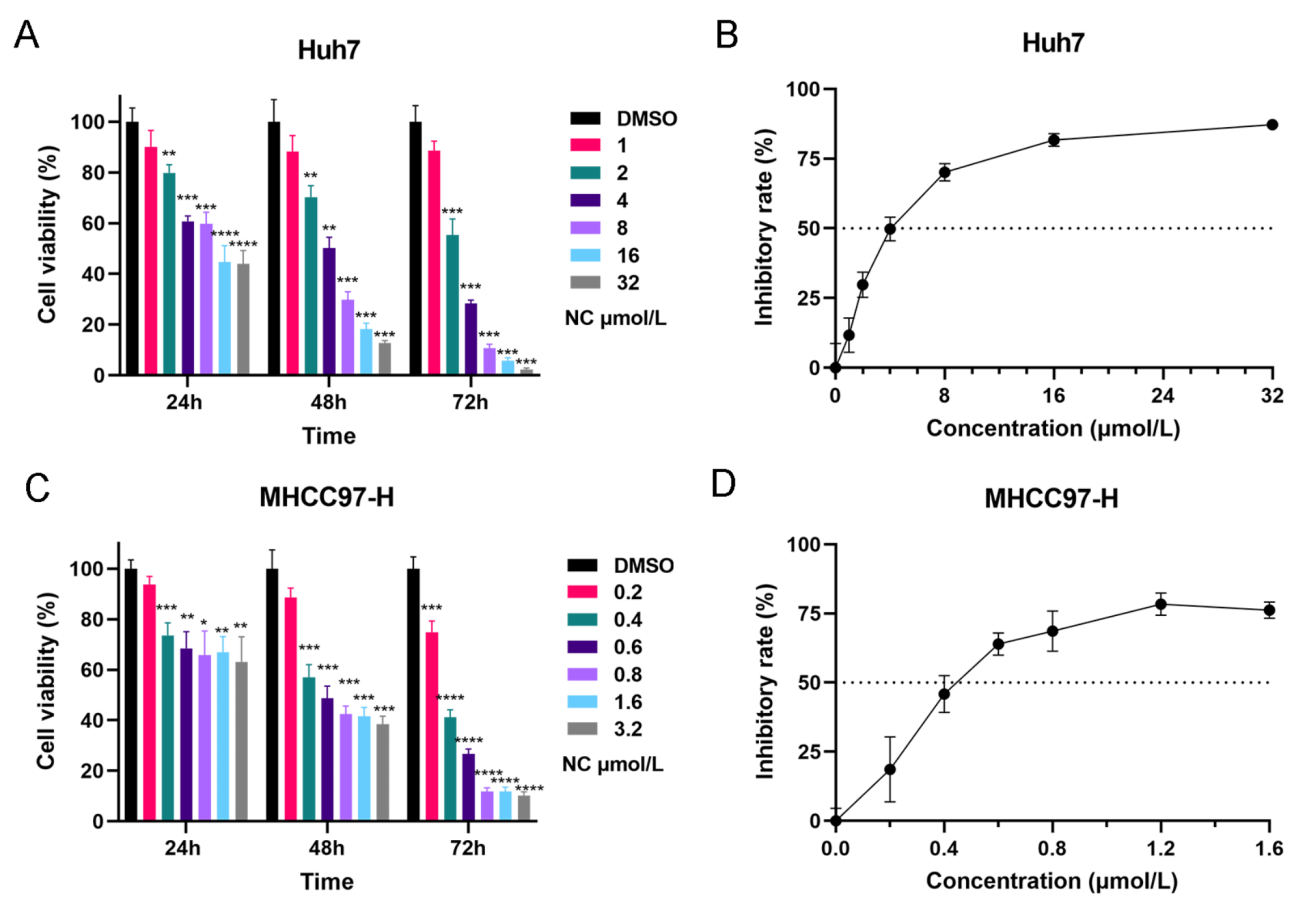
Inhibitory effect of nitidine chloride (NC) on the viability of HCC cells. **A** Cell viability of Huh7 following NC treatment at different concentrations for 24, 48 and 72 h; **B** Inhibition rate of Huh7 following NC treatment at different concentrations for 48 h; **C** Cell viability of MHCC97-H following NC treatment at different concentrations for 24, 48 and 72 h; **D** Inhibition rate of MHCC97-H following NC treatment at different concentrations for 48 h. DMSO, dimethyl sulfoxide. * *P* < 0.05, ** *P* < 0.01, *** *P* < 0.001, **** *P* < 0.0001 compared with the DMSO group

As shown in Fig. 2, NC exerted a growth inhibitory effect on both Huh7 and MHCC97-H cells in a time- and concentration-dependent manner. The 50% inhibitory concentration (IC50) of NC for Huh7 cells at 48 h was 4.240 (3.865–4.651) µmol/L, while for MHCC97-H cells, the IC50 was 0.4772 (0.4217–0.5349) µmol/L.

### NC inhibited the growth, metastasis and angiogenesis of HCC xenograft tumors in zebrafish

**Fig. 3:**
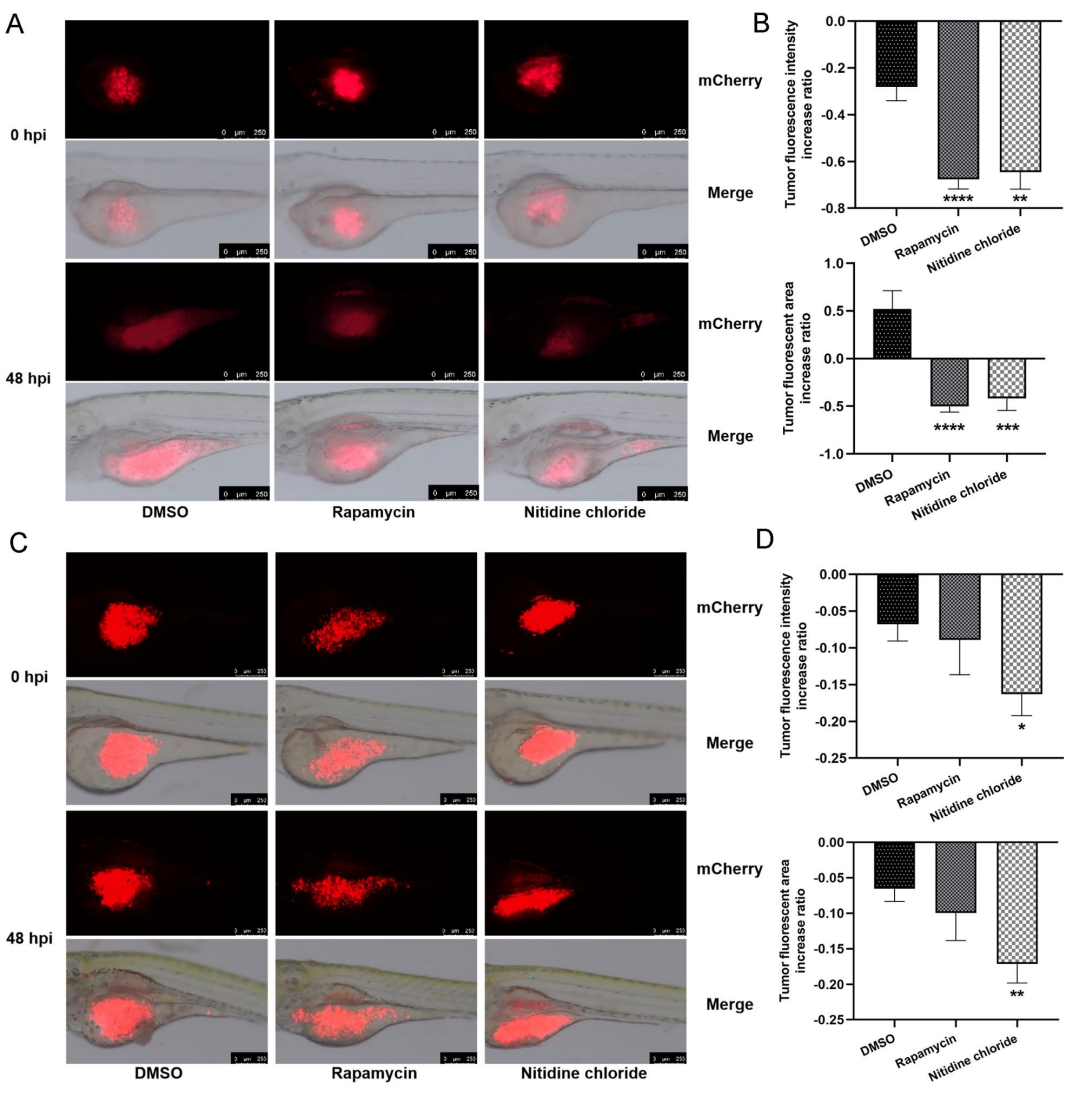
Inhibitory effect of nitidine chloride (NC) on the growth of hepatocellular carcinoma (HCC) zebrafish xenograft tumors. **A** Representative fluorescence images of the growth of human MHCC97-H xenografts in zebrafish inhibited by NC (3.0 ng) (Magnification: 80 ×, scale bar = 250 μm); **B** The inhibitory effect of NC on the MHCC97-H xenografted tumor growth in zebrafish, expressed by tumor fluorescence intensity and tumor fluorescent area; **C** Representative fluorescence images of the growth of the human Huh7 xenografts in zebrafish inhibited by NC (3.0 ng) (Magnification: 100 ×, scale bar = 250 μm); **D** The inhibitory effect of NC on the Huh7 xenografted tumor growth in zebrafish, expressed by tumor fluorescence intensity and tumor fluorescent area. hpi, hour post-injection; DMSO, solvent control group; Rapamycin, positive drug control group; Nitidine chloride, NC treated group; Data are displayed as mean ± SEM (*n* = 10). * *P* < 0.05, ** *P* < 0.01, *** *P* < 0.001, **** *P* < 0.0001 in comparison with the DMSO group

Wild-type AB and Tg (kdrl: EGFP) zebrafish were used to study the effect of NC on HCC growth in vivo. In comparison to the solvent control group (DMSO), the group treated with NC for both MHCC97-H (Fig. 3A–B) and Huh7 (Fig. 3C–D) zebrafish xenograft tumors displayed a noteworthy reduction in both tumor fluorescence intensity and tumor fluorescence area, indicating that NC could effectively inhibit the growth of HCC tumors.

**Fig. 4:**
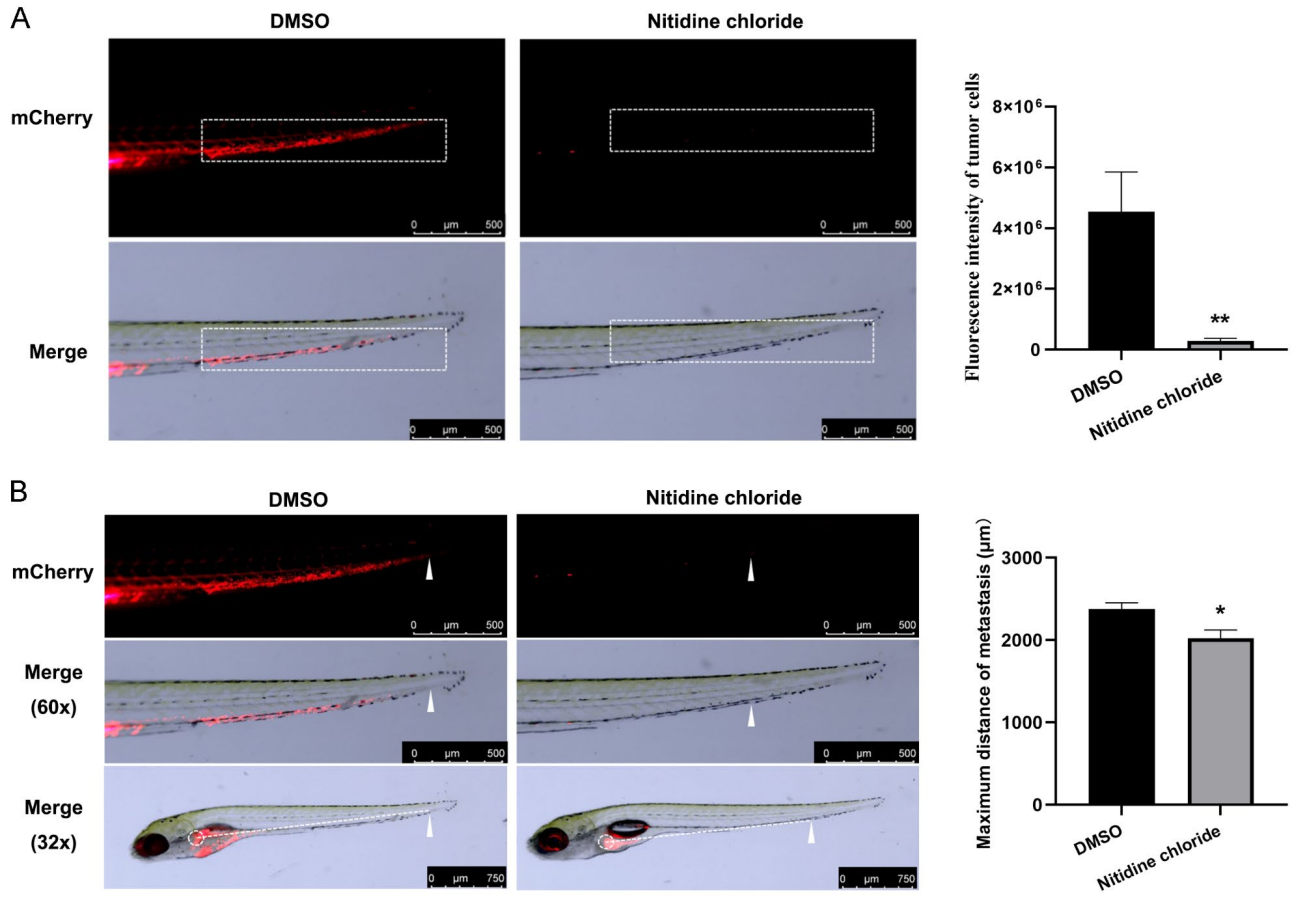
Inhibitory effect of nitidine chloride (NC) on the metastasis of hepatocellular carcinoma (HCC) zebrafish xenograft tumors. **A** The inhibitory effect of NC on the fluorescence intensity of distal metastatic tumor cells in MHCC97-H xenografted zebrafish. The red cell cluster within the box with white dotted line is the area used to measure fluorescent intensity (Magnification: 60 ×, scale bar = 500 μm); **B** The inhibitory effect of NC on maximum metastasis distance in MHCC97-H xenografted zebrafish. The white triangle represents the maximum metastatic tumor cells, the midpoint in the white dotted box is the starting point of the maximum metastatic distance, and the white dotted line represents the maximum metastatic distance of the tumor. Data are displayed as mean ± SEM (*n* = 6). * *P* < 0.05, ** *P* < 0.01 in comparison with the DMSO group

Wild-type AB zebrafish were also utilized to evaluate the effect of NC on MHCC97-H metastasis in vivo. In response to NC drug treatment, the fluorescence intensity of distal MHCC97-H metastatic tumors was significantly reduced (*P* < 0.01, Fig. 4A), along with the furthest metastatic distance (*P* < 0.05, Fig. 4B), suggesting NC could also significantly inhibit the metastasis of HCC cells in vivo.

**Fig. 5:**
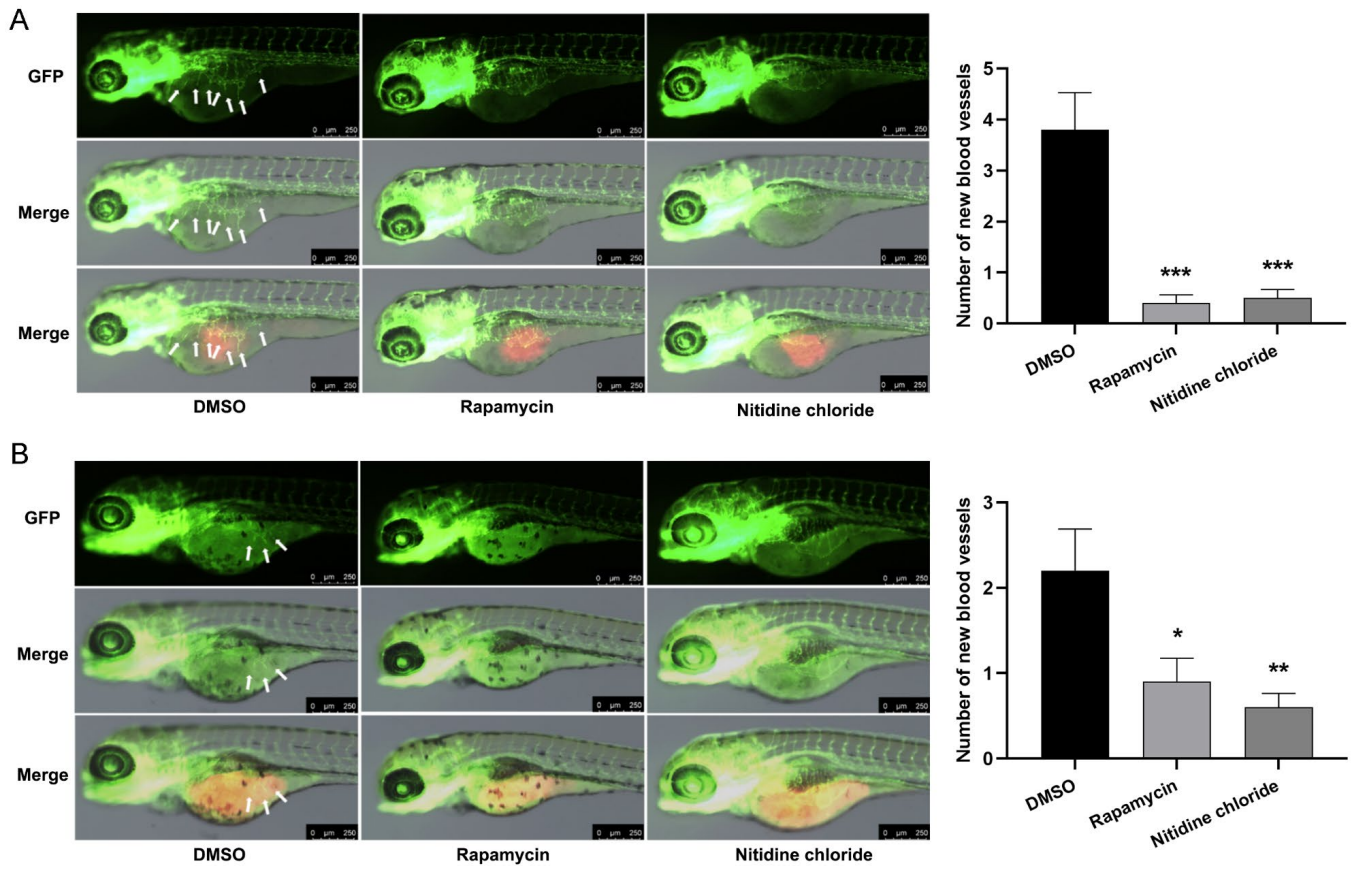
Inhibitory effect of nitidine chloride (NC) on the angiogenesis of hepatocellular carcinoma (HCC) zebrafish xenograft tumors. **A**,** B** Phenotype of subintestinal vascular growth and statistical analysis chart for MHCC97-H (**A**) and Huh7 (**B**). The white arrows indicate the newly formed blood vessels (Magnification: 80 ×, scale bar = 250 μm). DMSO, solvent control group; Rapamycin, positive drug control group; Nitidine chloride, NC treated group. Data are shown as mean ± SEM (*n* = 10). * *P* < 0.05, ** *P* < 0.01, *** *P* < 0.001 in comparison with the DMSO group

To further investigate the antitumor effect of NC in vivo, we employed transgenic zebrafish (flila: EGFP), a suitable model for researching tumor angiogenesis (Nathan and Kannan 2020), to explore how NC influences angiogenesis in HCC at a safe dosage of 3.0 ng. Compared with the solvent control group, a noteworthy reduction in subintestinal angiogenesis was observed in zebrafish carrying transplanted MHCC97-H or Huh7 tumors within the NC-treated group (*P* < 0.001, Fig. 5).

#### NC directly targeted and inhibited IGF2BP3 expression in HCC cells

**Fig. 6:**
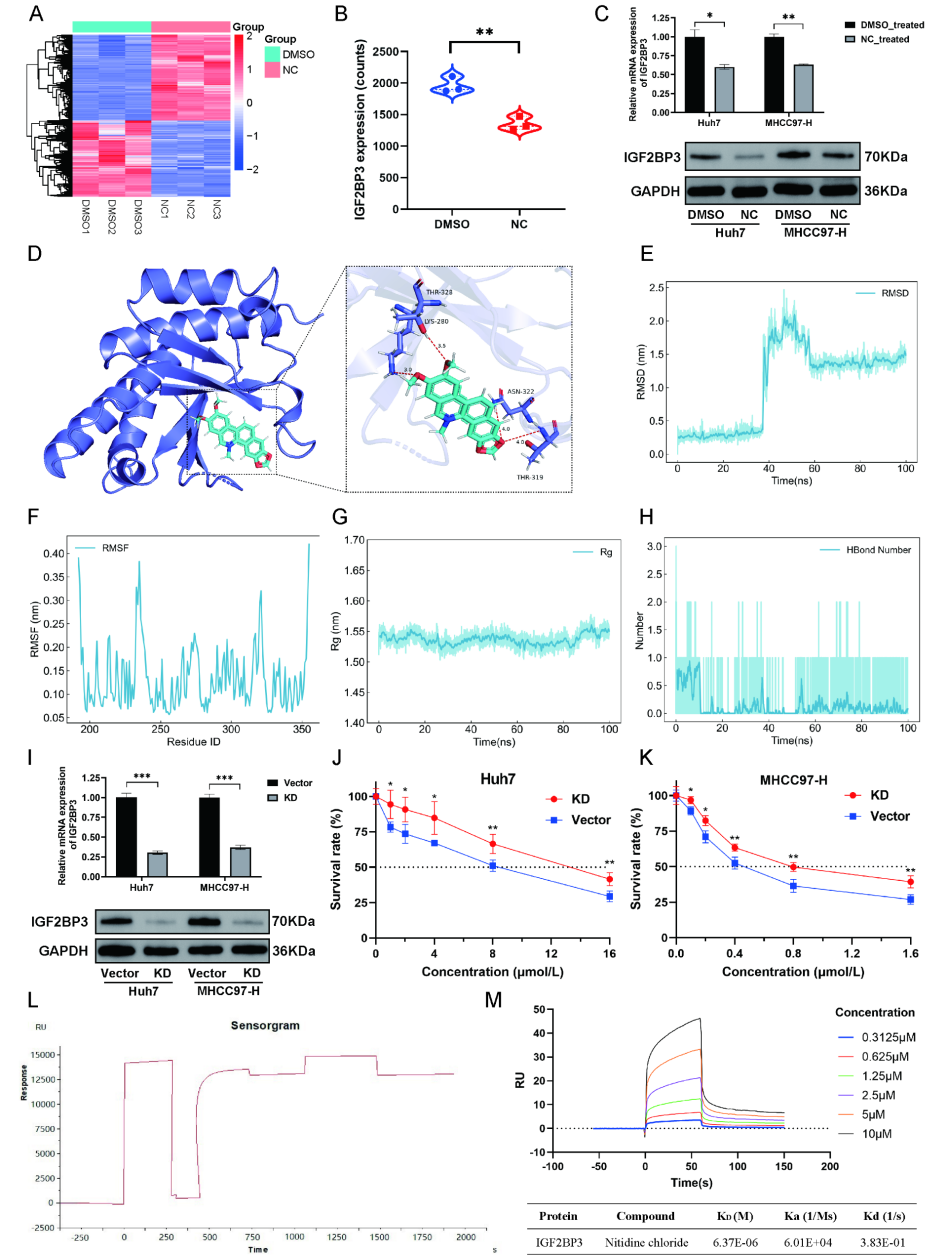
Nitidine chloride (NC) functioned by directly targeting and inhibiting IGF2BP3 expression in hepatocellular carcinoma (HCC) cells. **A** The heatmap of 8993 differently expressed genes (including 4226 down-regulated genes and 4767 up-regulated genes) between three pairs of control and NC-treated Huh7 cells; **B** Differential analysis of IGF2BP3 expression between three pairs of control and NC-treated Huh7 cells; **C** Expression level validation by real-time quantitative polymerase chain reaction (RT-qPCR) and western blotting (WB) in Huh7 and MHCC97-H cells treated and untreated with NC; **D** The binding model of IGF2BP3 and NC; **E–H** Molecular dynamics (MD) simulation analyses of IGF2BP3-NC complex including root mean square deviation (RMSD) (**E**), root mean square fluctuation (RMSF) (**F**), radius of gyration (Rg) (**G**), and hydrogen bond counts (**H**); **I** Knockdown of IGF2BP3 (IGF2BP3-KD) in Huh7 and MHCC97-H cells validated by RT-qPCR and WB; **J**,** K** Survival rate of IGF2BP3-KD and vector control HCC cells, including Huh7 (**J**) and MHCC97-H (**K**), following NC treatment at different concentrations for 48 h. **L** IGF2BP3 protein coupling map for surface plasmon resonance (SPR). **M** SPR analysis of the binding between IGF2PB3 and NC at the indicated concentrations. DMSO, dimethyl sulfoxide. KD, knockdown. K_D_, dissociation constant. Ka, association rate constant. Kd, dissociation rate constant. * *P* < 0.05, ** *P* < 0.01, *** *P* < 0.001

To explore the mechanism of NC against HCC, we treated Huh7 cells with 4 µmol/L of NC for 48 h and performed RNA-seq. A total of 8993 DEGs were obtained (Fig. 6A), with IGF2BP3 being a gene that showed significant downregulation in expression after NC treatment (log2FoldChange = -0.914, *P* < 0.01, Fig. 6B). Subsequent RT-qPCR and WB experiments further validated the reduction in both mRNA and protein levels of IGF2BP3 after NC treatment in both Huh7 and MHCC97-H cells (Fig. 6C).

To confirm whether NC interacts with IGF2BP3 directly or indirectly, we performed molecular docking analysis and MD simulation. The result of molecular docking showed that the binding site between NC and IGF2BP3 exhibited excellent spatial complementarity, and the formation of four hydrogen bonds significantly enhanced the stability of the binding conformation (Fig. 6D). The docking score between NC (ligand) and IGF2BP3 (receptor) was − 6.7 kcal/mol, indicating a stable binding interaction. MD simulation was further utilized to evaluate the binding stability. The RMSD analysis indicated that NC formed a stable binding with IGF2BP3 at 60 ns during the simulation and maintained this stability until the end of the 100 ns simulation (Fig. 6E). Throughout the simulation, the overall RMSF of the protein remained low (Fig. 6F), and the Radius of Gyration (Rg) curve for the protein remained relatively stable (Fig. 6G), suggesting the sampling was reliable. Additionally, the number of hydrogen bonds between NC and IGF2BP3 became stable after 60 ns and remained consistent till the end of the simulation, providing strong evidence for the robust interaction between NC and IGF2BP3 (Fig. 6H). These results suggested that the interaction between IGF2BP3 and NC was stable and reliable, and that NC might directly target IGF2BP3. We further performed experimental validation. The expression of IGF2BP3 in Huh7 and MHCC97-H was successfully silenced by lentiviral transfection (Fig. 6I). Then the cellular viability was detected under various concentrations of NC treatment by CCK8. We found that in both Huh7 (Fig. 6J) and MHCC97-H (Fig. 6K) cells, the IC50 of NC in the IGF2BP3 knockdown (IGF2BP3-KD) group was significantly higher than that of the vector control group (*P* < 0.05). This implies that compared to the control group, IGF2BP3-KD HCC cells exhibited reduced drug sensitivity to NC, providing evidence that the anti-HCC activity of NC is indeed through targeting IGF2BP3. SPR was subsequently employed to measure the real-time molecular interactions between IGF2BP3 and NC. IGF2BP3 protein was successfully immobilized on a CM5 Sensor Chip (Fig. 6L), and then IGF2BP3-NC affinity was measured at varying concentrations of NC. SPR analysis revealed a strong binding affinity between NC and IGF2BP3, with a dissociation constant (K_D_) value of 6.37 × 10^− 6^ M (Fig. 6M). These findings confirm that NC directly binds to IGF2BP3.

### High expression of IGF2BP3 indicated poor survival outcomes in HCC

In an extensive analysis encompassing 82 datasets from 39 different platforms, which included 3891 HCC and 3389 non-HCC samples, we observed a significant overexpression of IGF2BP3 mRNA in HCC tissues compared to normal liver tissues (SMD = 1.20, 95% CI = 1.05–1.36, Fig. S1). A further SROC analysis highlighted the potential of IGF2BP3 mRNA levels as a discriminatory biomarker between HCC and non-HCC conditions, demonstrating an area under the curve (AUC) value of 0.86 (95% CI = 0.83–0.89, Fig. S2). As depicted in Fig. S3, the IGF2BP3 protein was also found to be highly expressed in HCC tissues in comparison to normal liver tissues. Furthermore, we delved into the prognostic significance of IGF2BP3 expression in HCC. As shown in Fig. S4, our findings revealed a strong association between elevated levels of IGF2BP3 and adverse survival outcomes, including overall survival (HR = 1.66, 95% CI = 1.16–2.39, *P* = 0.0053), recurrence free survival (HR = 1.44, 95% CI = 1.03–2.01, *P* = 0.034), progress free survival (HR = 1.51, 95% CI = 1.13–2.02, *P* = 0.0055) and disease specific survival (HR = 1.68, 95% CI = 1.08–2.62, *P* = 0.02).

#### IGF2BP3 knockdown inhibited the proliferation, migration and invasion of HCC cells

**Fig. 7:**
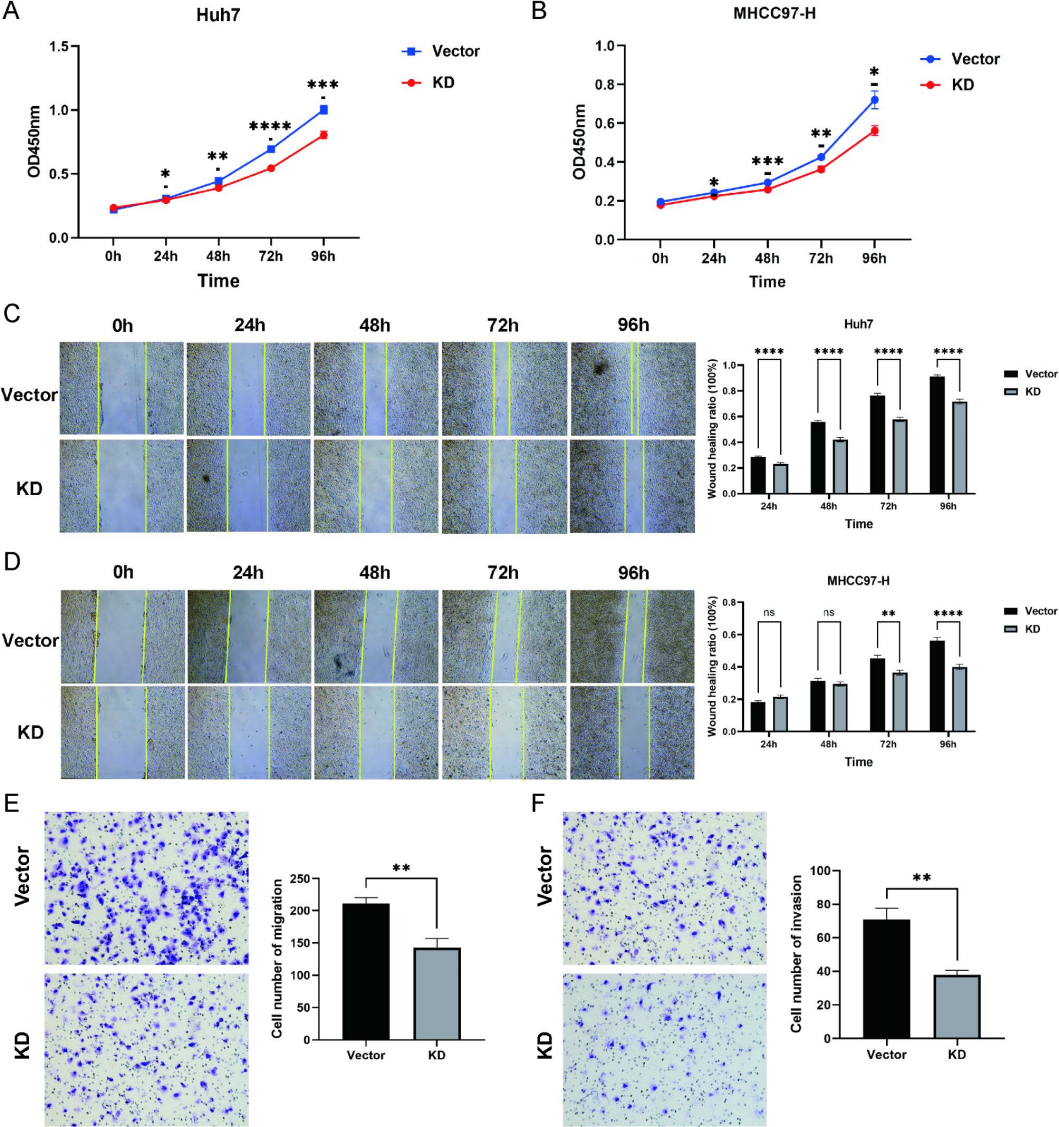
Effects of IGF2BP3 knockdown on HCC cell biological behaviors. **A–B** CCK8 assay detecting the proliferation ability of Huh7 (**A**) and MHCC97-H (**B**) cells after silencing IGF2BP3 expression. **C–D** Scratch assay detecting the migration ability of Huh7 (**C**) and MHCC97-H (**D**) cells after silencing IGF2BP3 expression (Magnification: 50 ×). **E–F** Transwell migration (**E**) and invasion (**F**) assays of Huh7 cells after silencing IGF2BP3 expression (Magnification: 100 ×). KD, knockdown. * *P* < 0.05, ** *P* < 0.01, *** *P* < 0.001, **** *P* < 0.0001

IGF2BP3 expression was successfully knocked down in Huh7 and MHCC97-H cells through lentiviral transfection (Fig. 6I). Compared with the control group, the IGF2BP3-KD group of Huh7 and MHCC97-H cells exhibited significantly reduced cell proliferation and wound healing rates (Fig. 7A–D). Transwell assays also demonstrated that cell migration and invasion abilities were markedly declined in the IGF2BP3-KD group of Huh7 cells (Fig. 7E–F).

#### Downstream target genes of IGF2BP3 inhibited by NC exhibited a strong correlation with metabolic processes

**Fig. 8:**
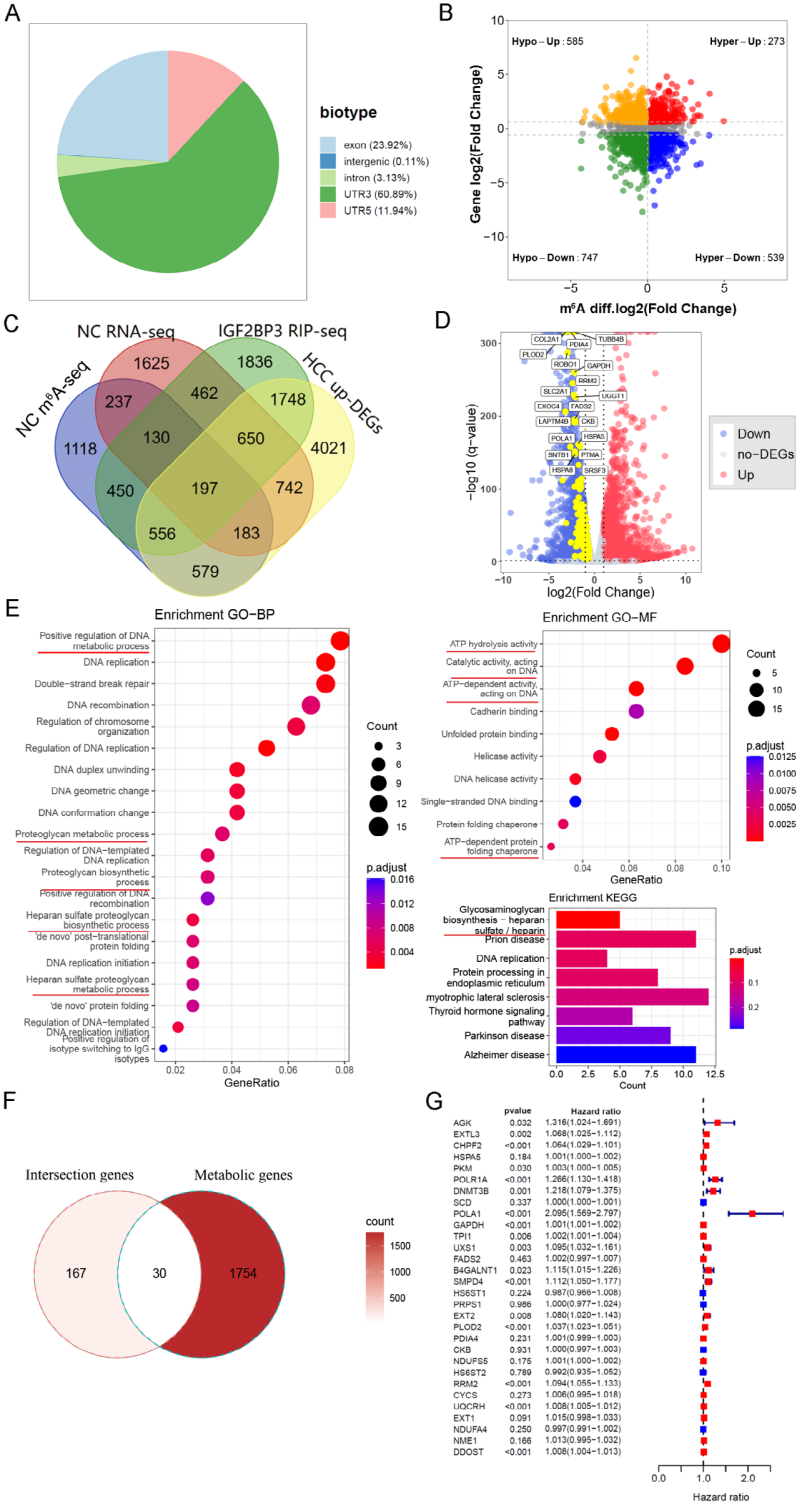
Identification and enrichment analysis of potential downstream target genes. **A** Distribution of differential m^6^A peaks in Huh7 cells following treatment with 4 µmol/L of Nitidine chloride (NC) for 48 h; **B** Four-quadrant plot depicting the integration analysis of the transcriptome and m^6^A profiling; **C** Venn diagram showing the overlapping genes obtained by taking intersections among several gene sets; **D** Volcano plot of the RNA-seq data following NC treatment, showcasing these 197 intersecting genes; **E** GO and KEGG enrichment analysis of these intersecting genes. BP: biological progress, MF: molecular function; **F** Venn diagram displaying metabolism-related target genes obtained by overlapping intersecting genes and metabolic genes; **G** Forest plot illustrating the regression analysis of metabolism-related target genes

Considering the established role of IGF2BP3 as an m^6^A reader, we postulated that NC might mediate its anti-HCC effects via the m^6^A pathway, a premise yet to be examined. Delving deeper into this potential link, we undertook an MeRIP-seq of Huh7 cells post 4 µmol/L NC administration. Our findings highlighted predominant differential m^6^A peaks within the UTR3 region (Fig. 8A). The differences in RNA and m^6^A levels of genes pre- and post-NC treatment were aggregated for subsequent analysis. Given that IGF2BP3 primarily functions to preserve gene stability, our analysis concentrated on genes that exhibited concurrent downregulation in both expression and m^6^A levels following NC treatment. Finally, 747 genes down-regulated at both mRNA and m^6^A levels following NC treatment were identified (Fig. 8B).

To further screen potential target genes regulated by IGF2BP3, we integrated the above 747 genes, 6,029 genes capable of directly binding with IGF2BP3 (derived from a RIP-seq dataset mentioned in the method part), and 8,676 genes identified as upregulated in HCC through SMD calculation. As a result, a set of 197 genes were identified (Fig. 8C–D). These genes were prospective downstream targets for NC to exert anti-HCC effects through the m^6^A mechanism, with their mRNA having the potential to directly bind to IGF2BP3. We conducted GO and KEGG functional enrichment analysis of these overlapping genes. Interestingly, these genes were enriched in multiple metabolic pathways, such as positive regulation of DNA metabolic process, proteoglycan metabolic process, proteoglycan biosynthetic process, glycosaminoglycan biosynthesis-heparan sulfate/heparin, and ATP activity-related pathways (Fig. 8E).

To further explore potential metabolic target genes of IGF2BP3 that may be down-regulated by NC, we took the intersection of these 197 intersection genes with 3463 metabolic-related genes derived from 114 metabolic pathways, resulting in 30 potential metabolic target genes of IGF2BP3 that are down-regulated by NC (Fig. 8F). Cox regression analysis was applied to these 30 genes, and the results revealed that most of them had HR values greater than 1, with p-values below 0.05 (Fig. 8G), suggesting that these genes could potentially serve as risk factors for HCC.

#### HCC patients with higher metabolic scores displayed poorer clinicopathological phenotypes and prognosis

ssGSEA analysis was performed based on the expression of the aforementioned 30 metabolic genes of IGF2BP3 to calculate a metabolic score for each HCC patient. Hierarchical clustering was used for clustering analysis, revealing that the optimal number of groupings was 2 (Fig. S5). Consequently, the cohort of 374 HCC patients was categorized into two distinct clusters: Cluster 1 with a low metabolic score and Cluster 2 with a high metabolic score (Fig. S6). The expression level of IGF2BP3 in Cluster 2 was significantly higher than that in Cluster 1 (Fig. S7). Additionally, there was a robust positive correlation between the metabolic scores and the expression levels of IGF2BP3 (Fig. S8).

To further explore the clinical significance of metabolic scoring and metabolic grouping in HCC, we investigated their relationship with clinicopathological parameters and patient prognosis. Fig. S9 depicted the overall clinical characteristics of metabolic subgroups and metabolic scores in patients with HCC. The upregulation of metabolic scores were remarkably associated with worse survival status, T stage, TNM stage, and histologic grade (Fig. S10). Analyzing the survival information from 370 HCC patients, it was observed that patients in Cluster 2 had a notably shorter overall survival compared to those in Cluster 1 (*P* = 0.016, Fig. S11). Univariate COX analysis revealed that higher metabolic scores predicted poorer survival outcomes in HCC (HR = 2.521, 95%CI = 1.333–4.769, *P* = 0.004, Fig. S12A). Multivariate COX analysis further suggested that metabolic score was an independent risk factor for HCC patients (HR = 2.229, 95%CI = 1.096–4.536, *P* = 0.027, Fig. S12B).

### IGF2BP3 regulated the expression of metabolic genes by enhancing their mRNA stability

**Fig. 9:**
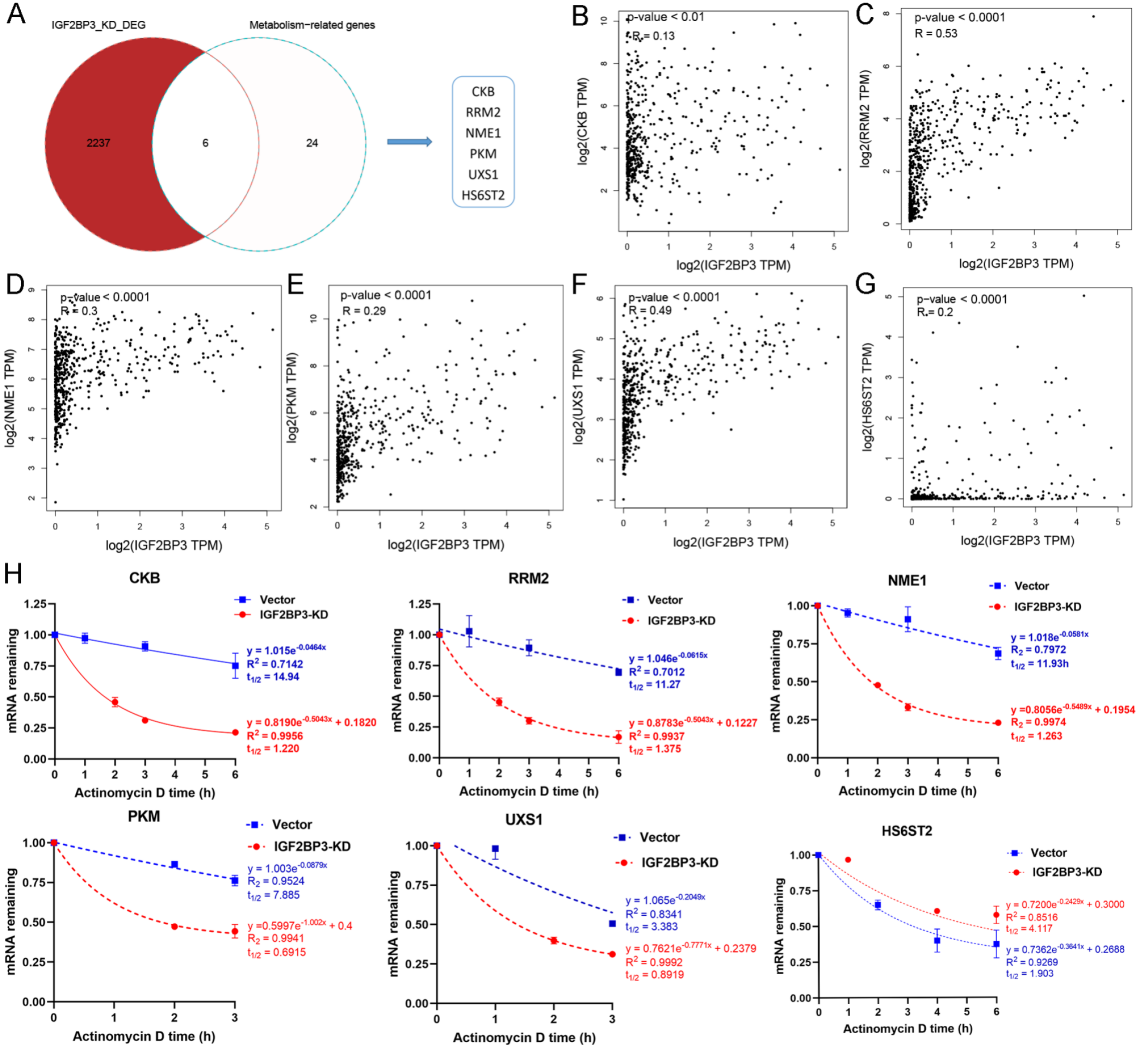
Identification and experimental validation of downstream metabolic genes regulated by IGF2BP3. **A** Venn diagram illustrating the overlap between differentially expressed genes upon IGF2BP3 knockdown and metabolism-related genes; **B–G** Correlation analysis between IGF2BP3 and the potential metabolic target genes by the GEPIA database, including CKB, RRM2, NME1, PKM, UXS1 and HS6ST2; **H** RNA stability assay of CKB, RRM2, NME1, PKM, UXS1 and HS6ST2 in Huh7 cells after IGF2BP3 knockdown. Transfected cells were treated with 5 µg/mL actinomycin D for 0 h, 1 h, 2 h, 3 h, 4 h or 6 h prior to RNA extraction

To delineate specific metabolic genes targeted by IGF2BP3, we intersected the above identified 30 genes with a set of 2243 genes that were found to be differentially downregulated following IGF2BP3 knockdown. This analysis yielded six intersecting genes: CKB, RRM2, NME1, PKM, UXS1, and HS6ST2 (Fig. 9A). Subsequent investigations into the relationship between IGF2BP3 and these genes were performed using the GEPIA database. The results revealed a statistically significant positive correlation between the expression of IGF2BP3 and all of the six genes (Fig. 9B–G). Recognizing the role of IGF2BP3 in enhancing mRNA stability of its downstream targets, we proceeded with RNA stability assays. The results demonstrated that IGF2BP3 knockdown significantly reduced the mRNA stability of CKB, RRM2, NME1, PKM, and UXS1, as evidenced by a marked decrease in their mRNA half-lives, while HS6ST2 was reversed (Fig. 9H). Additionally, NC treatment also reduced the mRNA stability of these five target genes (Fig. S13), further supporting the existence of the NC/IGF2BP3/m^6^A/metabolic gene regulatory axis.

## Discussion

NC, being a natural alkaloid, possesses various biological activities, including anti-tumor, anti-inflammatory, anti-malarial, anti-osteoporotic, and anti-rheumatic effects (Lu et al. 2022). An increasing number of studies suggest that NC is a promising antitumor agent that can exert tumor suppressive effects through multiple mechanisms (Cui et al. 2020). Our research team has been working on the mechanism of NC against HCC for a long time. To date, we have delineated a variety of potential mechanisms through which NC counteracts HCC, spanning molecular dimensions from mRNA, miRNA, lncRNA, to circRNA, and TF (Gao et al. 2019a, b, 2022; Liu et al. 2018; Xiong et al. 2019). Nevertheless, while these studies have primarily ventured into the potential mechanisms of NC against HCC at the gene expression level, a more profound elucidation of NC’s molecular mechanism remains to be explored. To delve deeper into this domain, the present study adopted a comprehensive approach, encompassing techniques such as RNA-seq, MeRIP-seq, RT-qPCR, WB, molecular docking, MD simulation, SPR, and both in vivo and in vitro assays, as well as lentiviral transfection and RNA stability experiments. Through these methods, we identified IGF2BP3 as a potential therapeutic target of NC treatment and revealed that NC could modulate metabolic pathways through targeting IGF2BP3 in an m^6^A-dependent manner. Our endeavors have pioneeringly paved the way for novel comprehension, illuminating the anti-cancer virtues of NC from an m^6^A epigenetic standpoint.

Zebrafish is a pivotal model organism for human disease research, characterized by its small size, large number of offspring, short life cycle, transparent embryos, in vitro embryonic development, and high homology with human genes. It is widely employed in the fields of tumor formation, invasion, metastasis, angiogenesis, and drug discovery (Letrado et al. 2018; Liu and Leach 2011; MacRae and Peterson 2015). Here, we established zebrafish xenograft tumor models for HCC by microinjecting HCC cells into zebrafish embryos. Employing this model, we probed the therapeutic efficacy of NC against HCC. Our observations highlighted that NC notably curtailed the growth, metastasis, and angiogenesis of HCC in vivo. These insights rectify the constraints of our preceding investigations, which were limited by the absence of in vivo assays, underscoring the promising clinical applicability of NC.

IGF2BP3 is an m^6^A reader that exerts oncogenic effects in various tumors (Sun et al. 2022). Previous research has highlighted its overexpression in HCC, associating it with an unfavorable prognosis and endorsing its role in promoting HCC growth and metastasis (Jeng et al. 2008; Jiang et al. 2020; Li et al. 2022), which aligns with the findings of our present investigation. The function of IGF2BP3 primarily revolves around recognizing m^6^A binding sites on target genes, thereby maintaining their stability (Huang et al. 2018). In this study, we performed RNA-seq and MeRIP-seq on NC-treated Huh7 cells. The results demonstrated a significant downregulation of IGF2BP3 following NC treatment, and the entire gene profile exhibited extensive alteration in m^6^A levels. Meanwhile, RT-qPCR and WB experiments verified that NC downregulated the expression of IGF2BP3. To investigate whether NC directly targeted IGF2BP3, we employed a combination of molecular docking, MR simulation, CCK8 off-targeting assay, and SPR for further confirmation. Molecular docking analysis indicated that NC could form a stable complex with IGF2BP3. MD simulations further confirmed this interaction, showing that a stable complex was formed between NC and IGF2BP3, and the number of hydrogen bonds was stabilized during the 100 ns simulation. CCK8 off-target experiments supported that NC targets IGF2BP3, although whether NC directly binds to IGF2BP3 still requires additional experimental validation. SPR is a well-established technique for studying molecular-protein interactions, where the protein is immobilized on the surface of a biosensor, and small molecules flow through the surface of the sensor chip. When a molecule binds to the protein on the surface, a change in refractive index occurs, reflecting the molecular interaction (Guerreiro et al. 2014; Sun et al. 2023). In this study, through SPR, we further confirmed a strong binding affinity between NC and IGF2BP3. In summary, by integrating multiple experimental approaches, we demonstrated that IGF2BP3 is a direct target for NC’s anti-HCC action. This suggests that NC might counteract HCC by directly targeting IGF2BP3, thereby modulating the m^6^A modification of its downstream target genes.

To further investigate the downstream mechanism of NC inhibiting HCC progression by targeting IGF2BP3, we conducted an intersection analysis of genes that can directly bind to IGF2BP3, genes with decreased mRNA and m^6^A levels after NC treatment, and genes with differentially high expression in HCC. This analysis resulted in 197 genes. By conducting enrichment analysis, we discovered that these genes are primarily enriched in metabolic-related pathways. This implies that by targeting IGF2BP3, NC can further influence cellular metabolic activities to exert its anti-HCC effects. The relationship between NC and metabolic processes has ever been reported. Mao et al. (Mao et al. 2019) demonstrated that NC induces inhibition of CYP1 enzymes (including CYP1A1 and CYP1B1) and leads to alterations in estradiol metabolism, which is primarily achieved by influencing the metabolic pathway of 2-hydroxylation. In our previous studies, we also reported that acid phosphatase type 6, functioning as a regulatory mitochondrial lipid phosphatase in lipid metabolism, could serve as a potential target for the anti-HCC effects of NC (Gao et al. 2022). Recently, increasing numbers of studies have highlighted that m^6^A plays a substantial role in the regulation of tumor metabolism (An and Duan 2022; Li et al. 2020). Cui et al. (Cui et al. 2023) reported that circFOXK2 together with IGF2BP3 accelerated aerobic glycolysis in oral squamous cell carcinoma by facilitating GLUT1 mRNA stabilization in an m^6^A-dependent manner. Additionally, Lin et al. (Lin et al. 2023) found that metabolic reprogramming mediated by the IGF2BP3-COX6B2 axis plays a key role in the development of acquired resistance to EGFR inhibitors in lung cancer. Based on the results of the enrichment analysis and the existing literature, we propose the following hypothesis: NC exerts its effects by targeting IGF2BP3 to regulate specific metabolic genes in the metabolic pathway in an m^6^A-dependent manner.

To further identify metabolism-related target genes of IGF2BP3, we subsequently refined the previously intersected gene set by intersecting it with metabolic genes. Based on these metabolism-related genes, we calculated the metabolic score for each HCC patient. We found that the metabolic score was significantly correlated with status, T stage, pathological stage, and histologic grade. Based on the metabolic score, HCC patients could be clustered into high and low metabolic groups, and the high metabolic group had a shorter overall survival compared to the low metabolic group. Further COX regression analysis suggested that metabolic score was an independent risk factor for the prognosis of HCC patients. These findings reveal that the IGF2BP3-related metabolic score can serve as a predictor of HCC prognosis, and grouping based on metabolic scores could be an effective strategy for predicting the survival of individuals with HCC.

Furthermore, six metabolic-related genes (CKB, RRM2, PKM, UXS1, NME1, and HS6ST2) that may be directed targeted by IGFBP3 were obtained. RNA stability experiment showed that compared to the control group, the mRNA half-life of CKB, RRM2, NME1, PKM, and UXS1 were dramatically reduced in both IGF2BP3 knockdown and NC-treated cells. According to the table of 114 metabolic pathways compiled by Rosario et al. (Rosario et al. 2018), CKB is involved in arginine and proline metabolism. RRM2 and NME1 are associated with pyrimidine metabolism, purine metabolism, pyrimidine biosynthesis, and glutathione metabolism. PKM is linked to pyruvate metabolism, purine Metabolism, glycolysis. UXS1 is connected to amino sugar and nucleotide sugar metabolism. These results imply that IGF2BP3 may regulate CKB, RRM2, NME1, PKM, and UXS1 in an m^6^A-dependent manner, thereby influencing metabolic processes, including lipid metabolism, protein metabolism, glucose metabolism, and nucleotide metabolism. Naturally, further experimental verifications need to be done.

Despite these comprehensive findings, certain aspects necessitate further investigation. Specifically, we lack other concrete evidence demonstrating NC’s direct binding to IGF2BP3 and whether IGF2BP3 indeed modifies downstream genes via m^6^A modification. Rigorous methods such as streptavidin-biotin affinity pull-down assay, immunoprecipitation, and MeRIP-qPCR experiments are required to address these inquiries. Additionally, animal experiments to verify that NC inhibits HCC progression by inhibiting IGF2BP3 also need to be performed. We will continue to refine these aspects in further studies.

## Conclusions

In conclusion, in this study, we proved that NC can exert antitumor effects by targeting IGF2BP3 via the m^6^A mechanism and identified a serials of metabolic-related target genes, among which CKB, RRM2, NME1, PKM, and UXS1 were confirmed as downstream targets of IGF2BP3. These findings provide a deeper investigation and insight into the anti-HCC mechanism of NC. Furthermore, the corresponding in vivo experiments in zebrafish also contribute to enhance the potential for the clinical translational application of NC.

## Electronic supplementary material

Below is the link to the electronic supplementary material.

Supplementary Material 1

Supplementary Material 2

## Data Availability

The data used to support the findings of this study are available from the corresponding author upon reasonable request.

## Abbreviations

HCC: Hepatocellular carcinoma
NC: Nitidine chloride
m^6^A: N6-methyladenosine
MeRIP-seq: RNA immunoprecipitation sequencing
RIP-seq: RNA immunoprecipitation sequencing
RT-qPCR: Real-time quantitative polymerase chain reaction Polymerase chain reaction
WB: Western blotting
MD: Molecular dynamics
SPR: Surface plasmon resonance
KD: Knock down
K_D_: Dissociation constant
DMSO: Dimethyl sulfoxide
CCK8: Cell counting kit-8
DEG: Differentially expressed gene
GEO: Gene Expression Omnibus
TCGA: The Cancer Genome Atlas
GTEx: Genotype-Tissue Expression
SMD: Standardized mean difference
CI: Confidence interval
SROC: Summary receiver operating characteristic
HR: Hazard ratio
GO: Gene Ontology
KEGG: Kyoto Encyclopedia of Genes and Genomes
ssGSEA: Single-sample gene set enrichment analysis
IC50: 50% inhibitory concentration
AUC: Area under the curve
