# Supplementary material for: Nitidine chloride inhibits the progression of hepatocellular carcinoma by suppressing IGF2BP3 and modulates metabolic pathways in an m6A-dependent manner
Source: Mol Med. 2025 Feb 5;31:47. doi: 10.1186/s10020-025-01095-8 (PMC11796242; doi:10.1186/s10020-025-01095-8)
Supplement: Supplementary file 1 — Supplementary Material 1 [file 10020_2025_1095_MOESM1_ESM.docx]

**Supplementary material**

**Table S1. The primers used in this study.**

| Name | Forward (5'-3') | Reverse (5'-3') |
| --- | --- | --- |
| IGF2BP3 | GCTCTATCAGTCGGTGCCATCATC | GCCTTGAACTGAGCCTCTGGTG |
| CKB | GGCCGATACTACGCGCTCA | ACCCACACCAGGAAGGTCT |
| RRM2 | GGCCGATACTACGCGCTCA | ACCCACACCAGGAAGGTCT |
| PKM | ATGTCGAAGCCCCATAGTGAA | TGGGTGGTGAATCAATGTCCA |
| UXS1 | ACATGAAGCAGGAAGGCGTG | CGTACTGGAACGCCCTTGTC |
| NME1 | CGGGGTCTTGTGGGAGAGATT | TGCATGTATTTCACCAGGCCG |
| HS6ST2 | CCCGAAGCAGAACTCAGGCA | GCCATCTTTTCAGTGGCGCTT |
| GAPDH | AATCAAGTGGGGCGATGCTG | GCAAATGAGCCCCAGCCTTC |
| B2M | TGAAGCTGACAGCATTCGG | CTGCTGGATGACGTGAGTAAA |


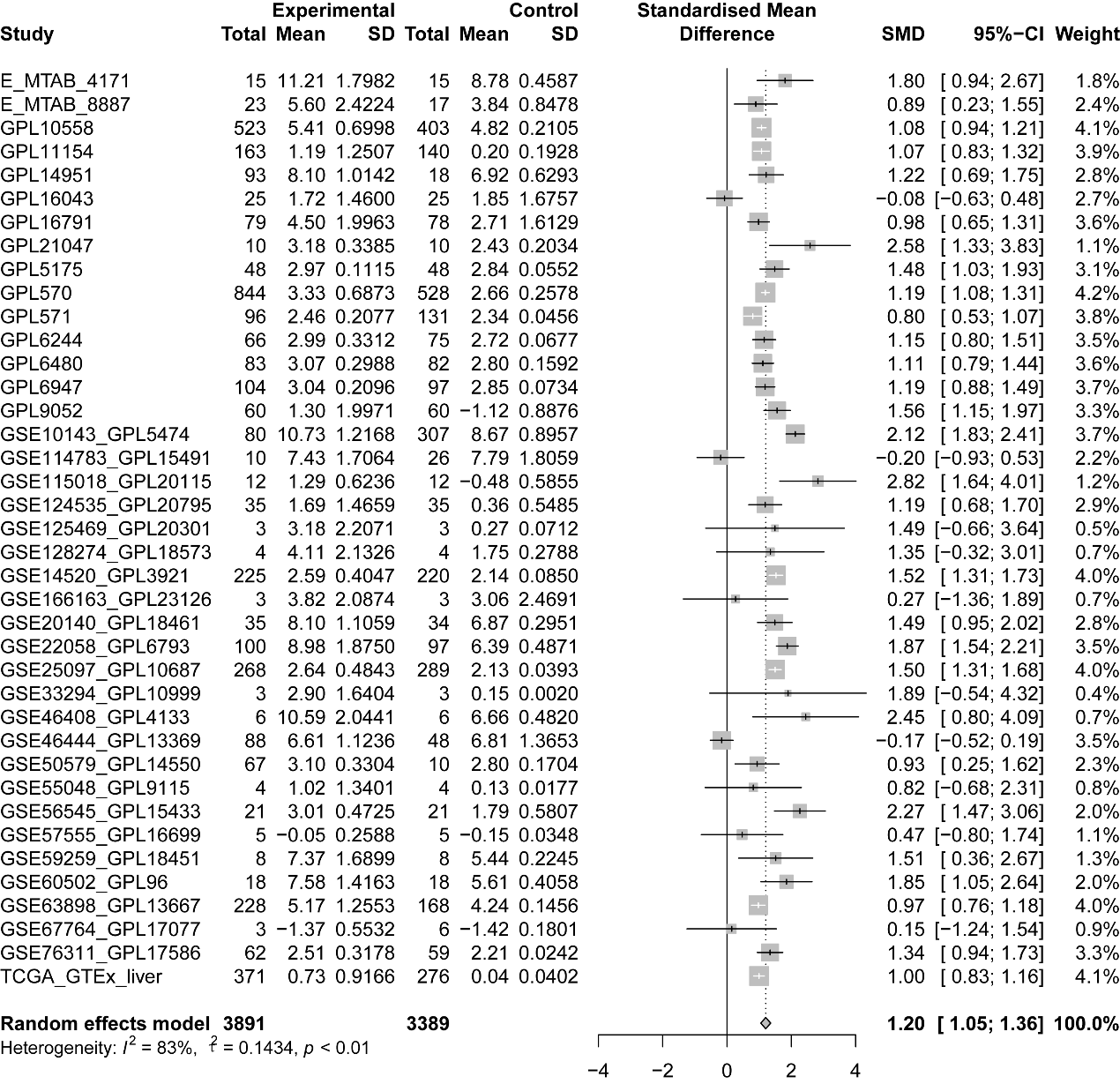


**Fig. S1** Forest plot depicting IGF2BP3 mRNA expression in HCC based on 66 datasets from 34 platforms.

**
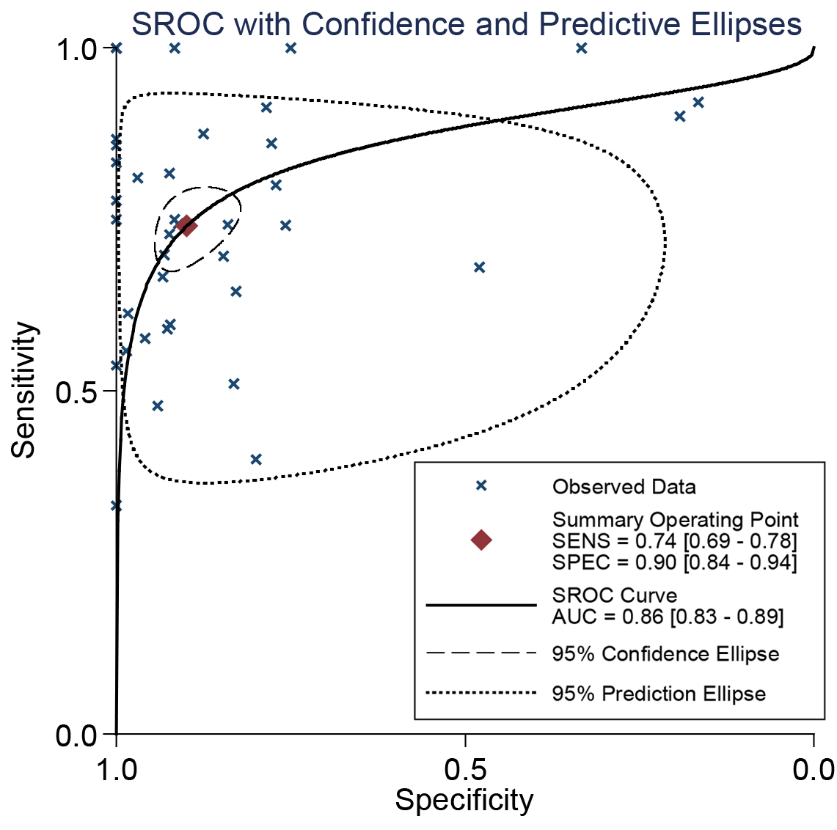
**

**Fig. S2** Summary receiver operating characteristic (SROC) curve showing the ability of IGF2BP3 to discriminate HCC samples from normal controls.

**
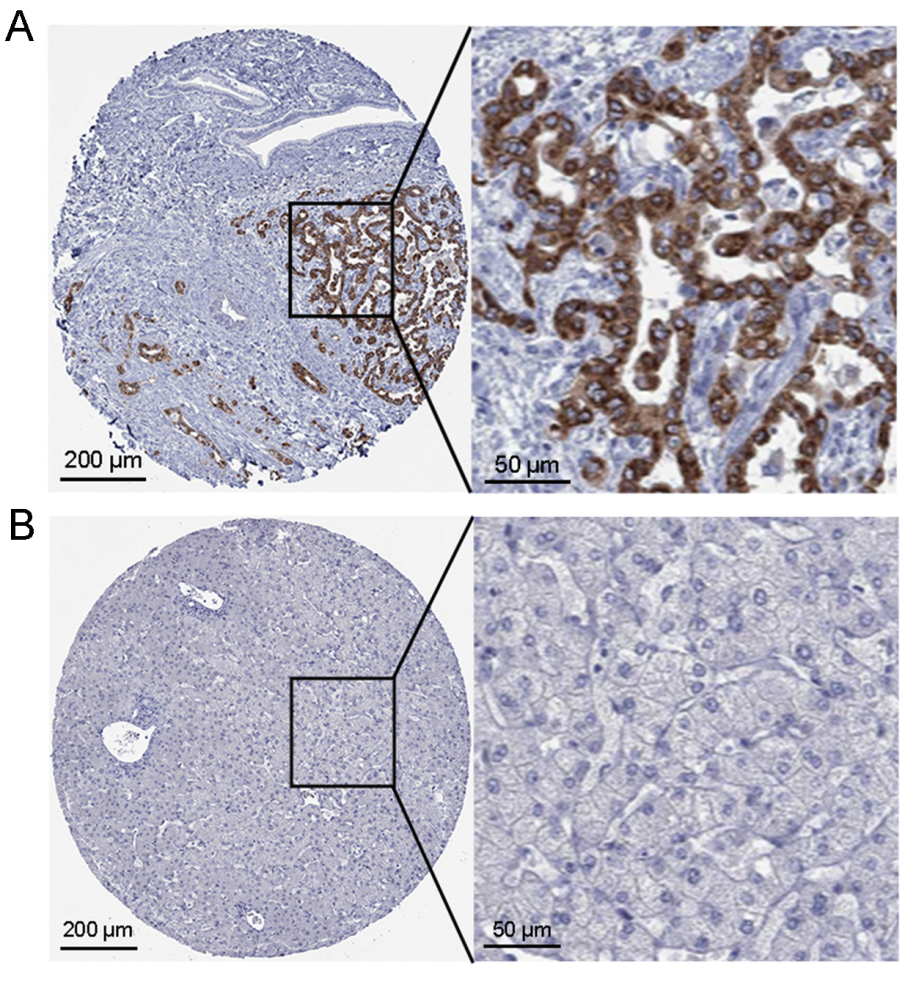
**

**Fig. S3** The immunohistochemical images of IGF2BP3 in HCC tissues (A) and normal liver tissues (B) from the Human Protein Atlas database.

**
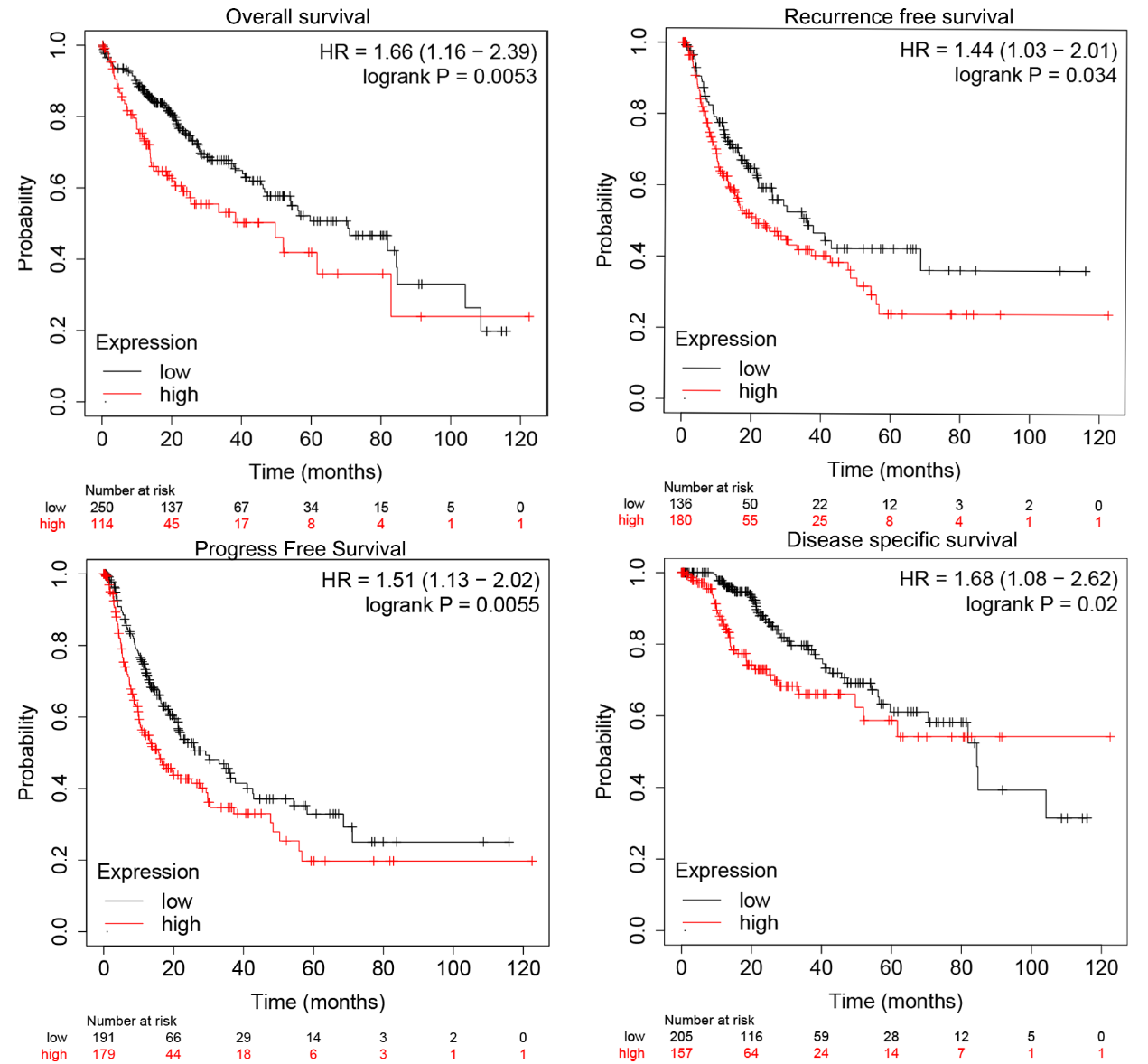
**

**Fig. S4** Relationship of IGF2BP3 mRNA expression with overall survival, recurrence free survival, progress free survival and disease specific survival by the Kaplan-Meier Plotter database. HR, hazard ratio.


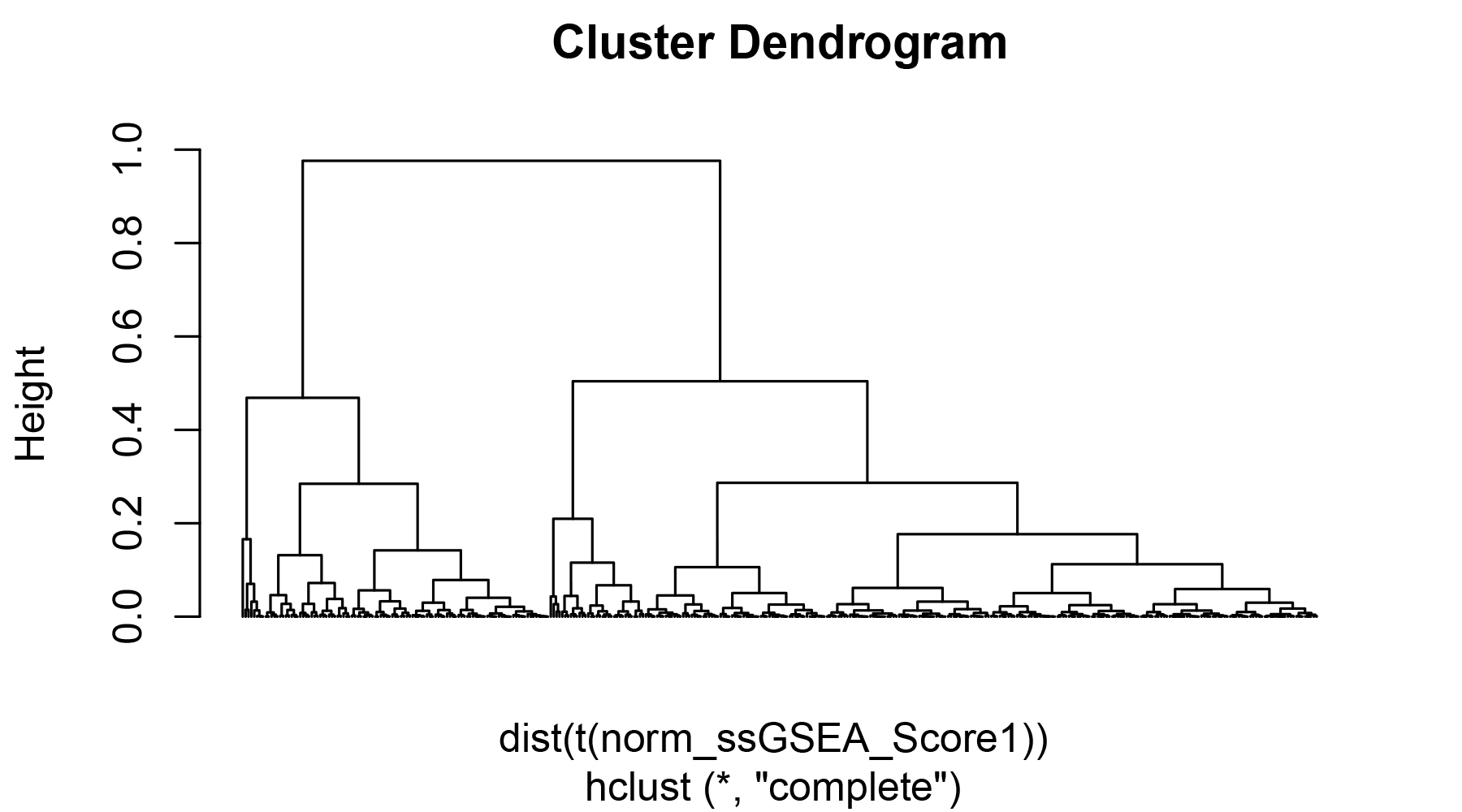


**Fig. S5** Hierarchical clustering of HCC patients based on metabolic scores.


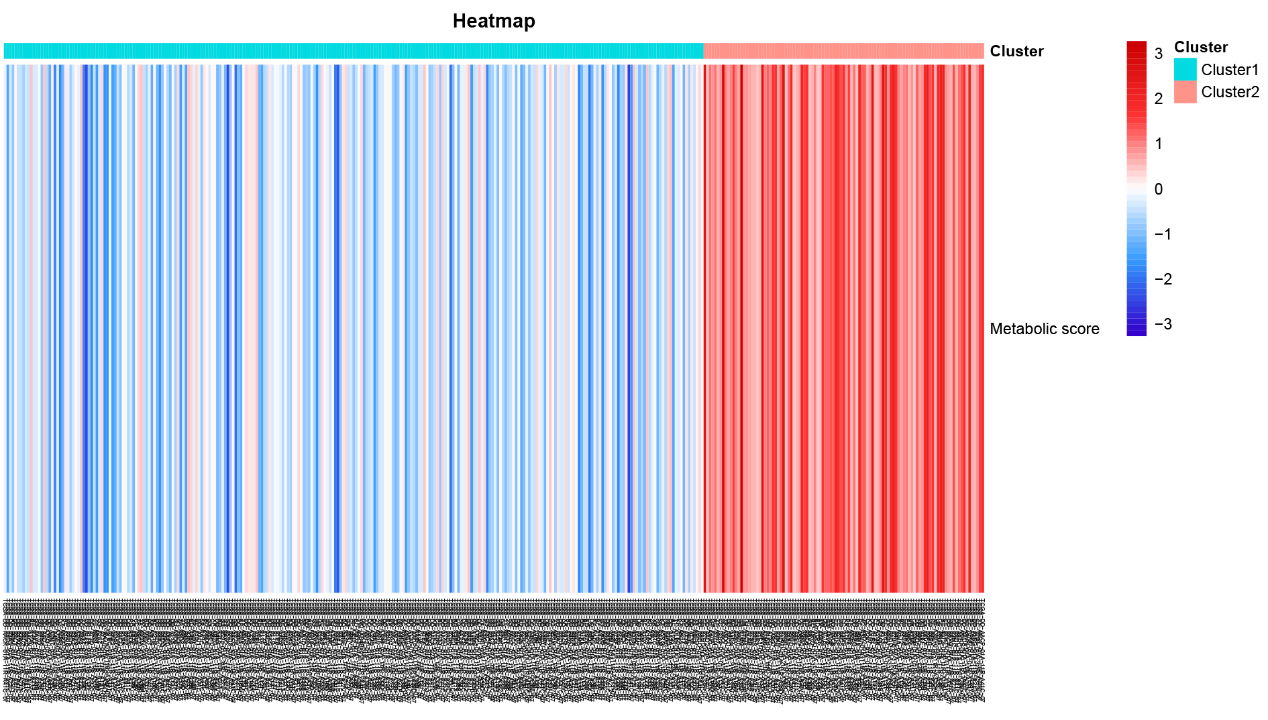


**Fig. S6** The heatmap of clustering results based on metabolic scores. Cluster 1, HCC patients with low metabolic scores; Cluster 2, HCC patients with high metabolic scores.


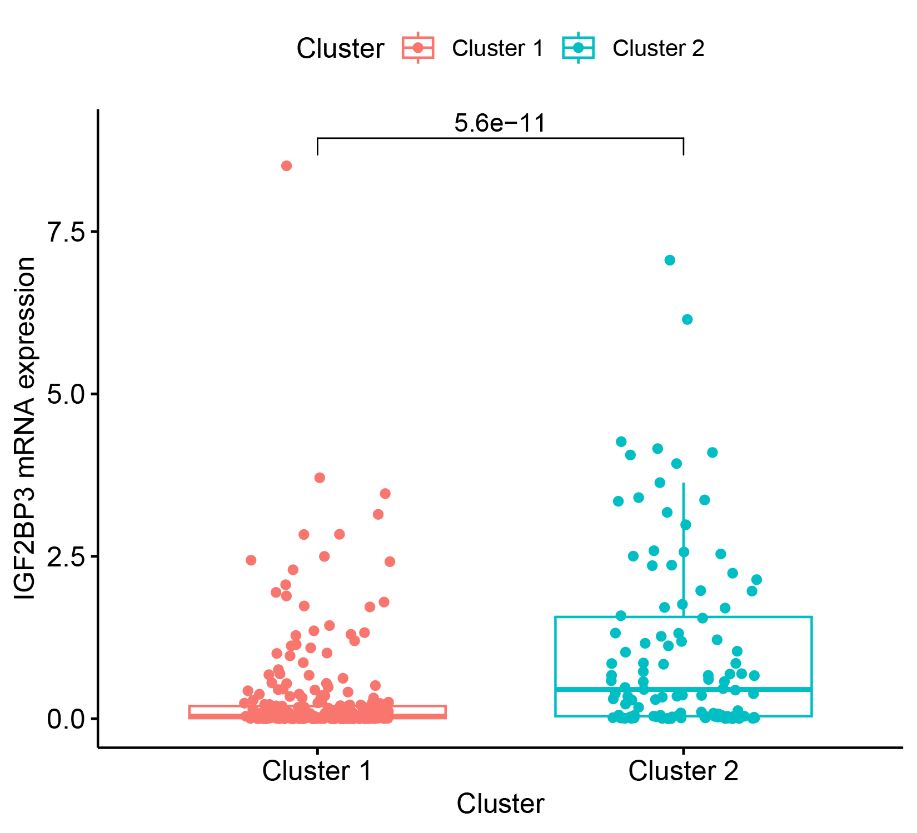


**Fig. S7** Box plot illustrating the expression levels of IGF2BP3 in HCC patients with high and low metabolic scores. Cluster 1, HCC patients with low metabolic scores; Cluster 2, HCC patients with high metabolic scores.


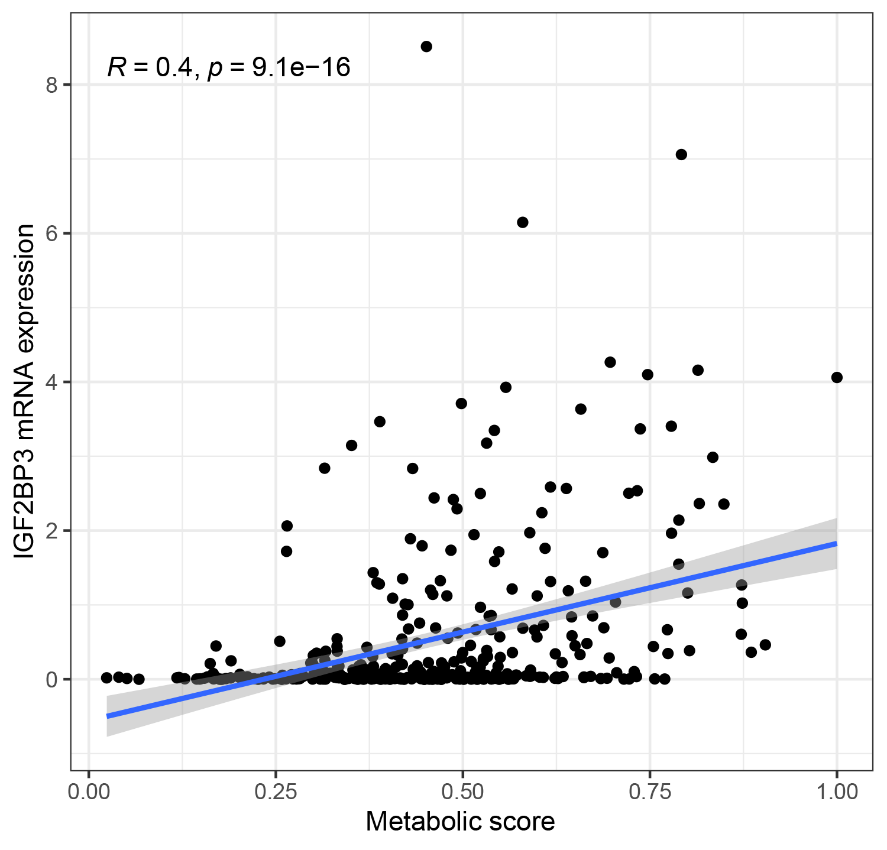


**Fig. S8** Correlation analysis between metabolic score and IGF2BP3 mRNA expression.


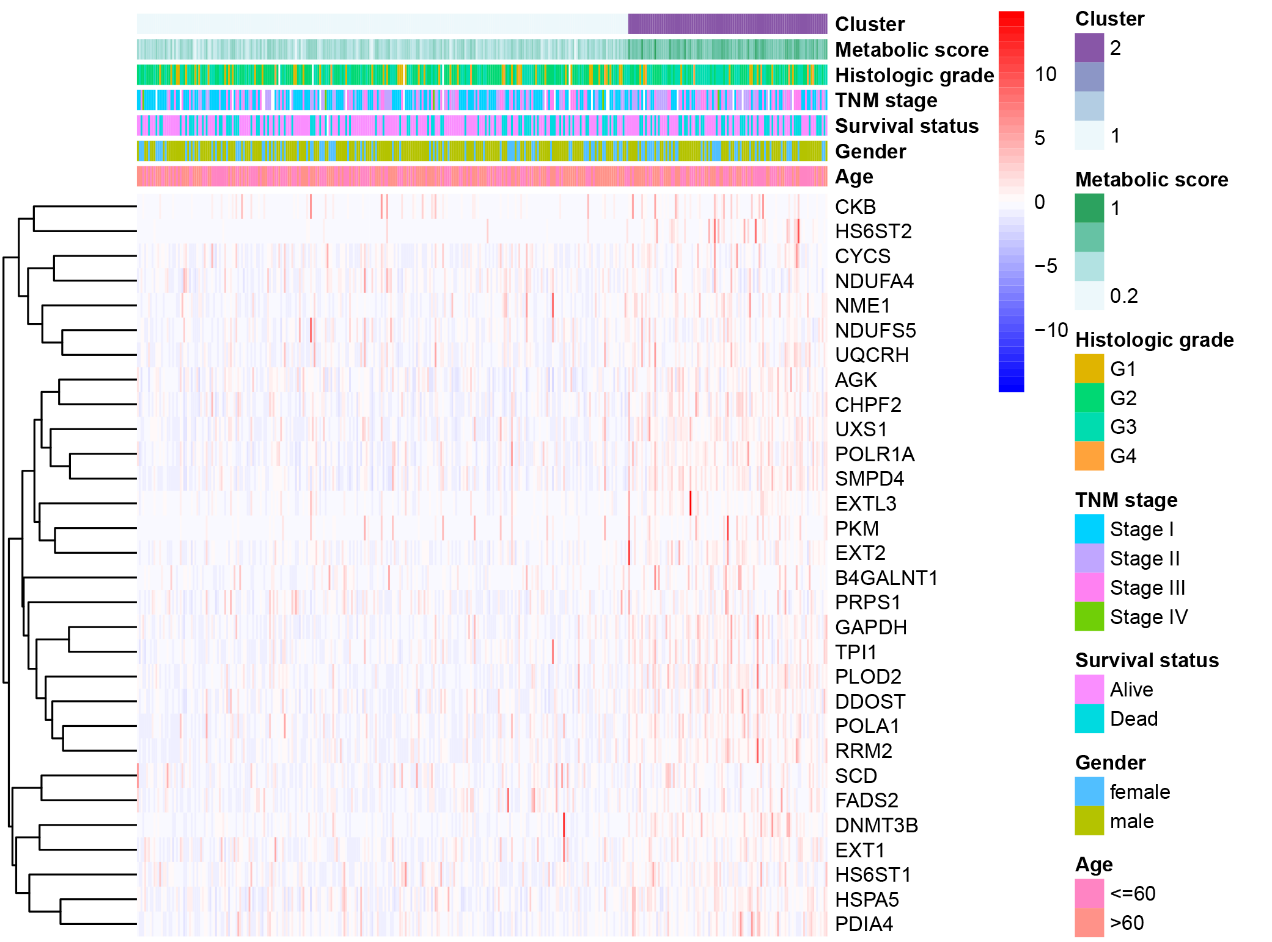
 **Fig. S9** The heatmap depicting the overall clinical characteristics of HCC patients clustered by metabolic scores. Cluster 1, HCC patients with low metabolic scores; Cluster 2, HCC patients with high metabolic scores.


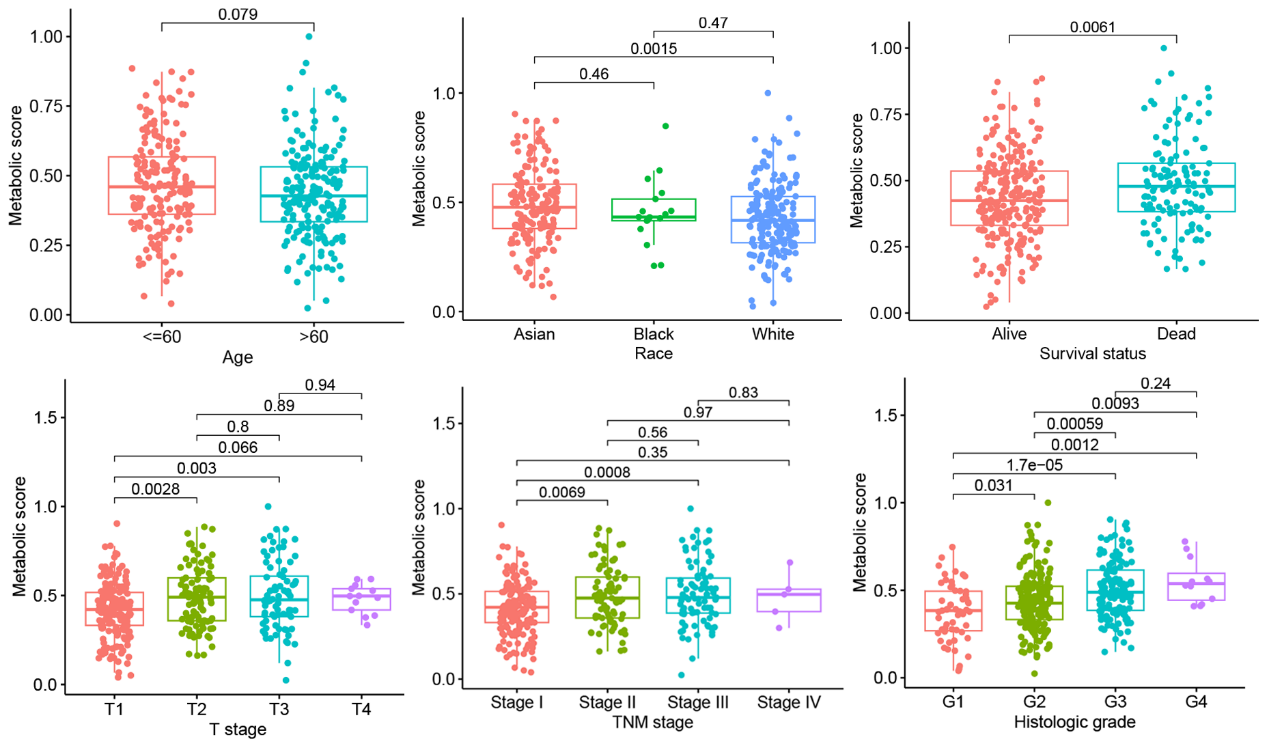


**Fig. S10** Relationship between metabolic scores and clinicopathologic parameters, including age, race, survival status, T stage, TNM stage and Histologic grade.

**
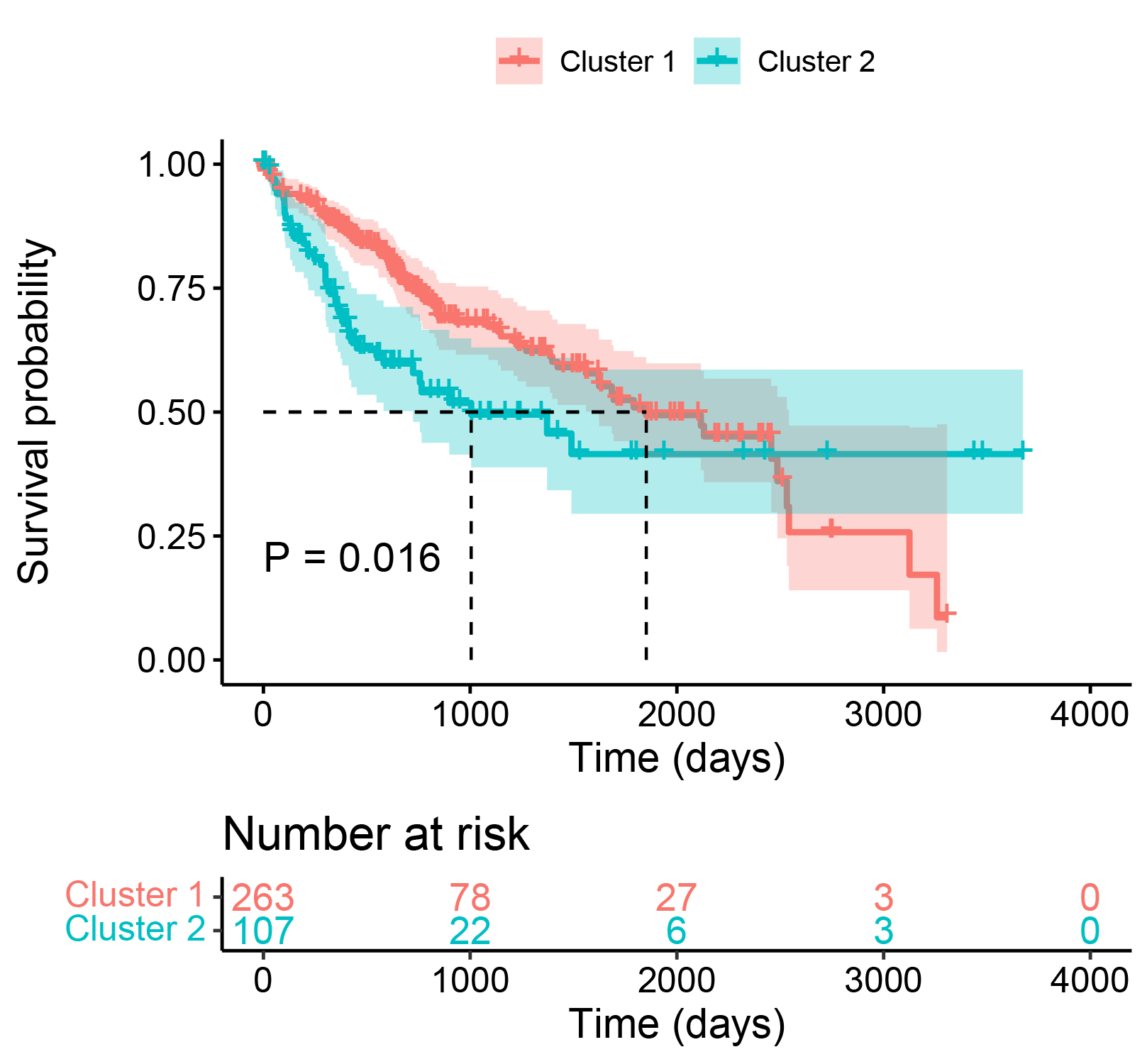
**

**Fig. S11** Kaplan-Meier overall survival analysis of HCC patients with high and low metabolic scores. Cluster 1, HCC patients with low metabolic scores; Cluster 2, HCC patients with high metabolic scores.

**
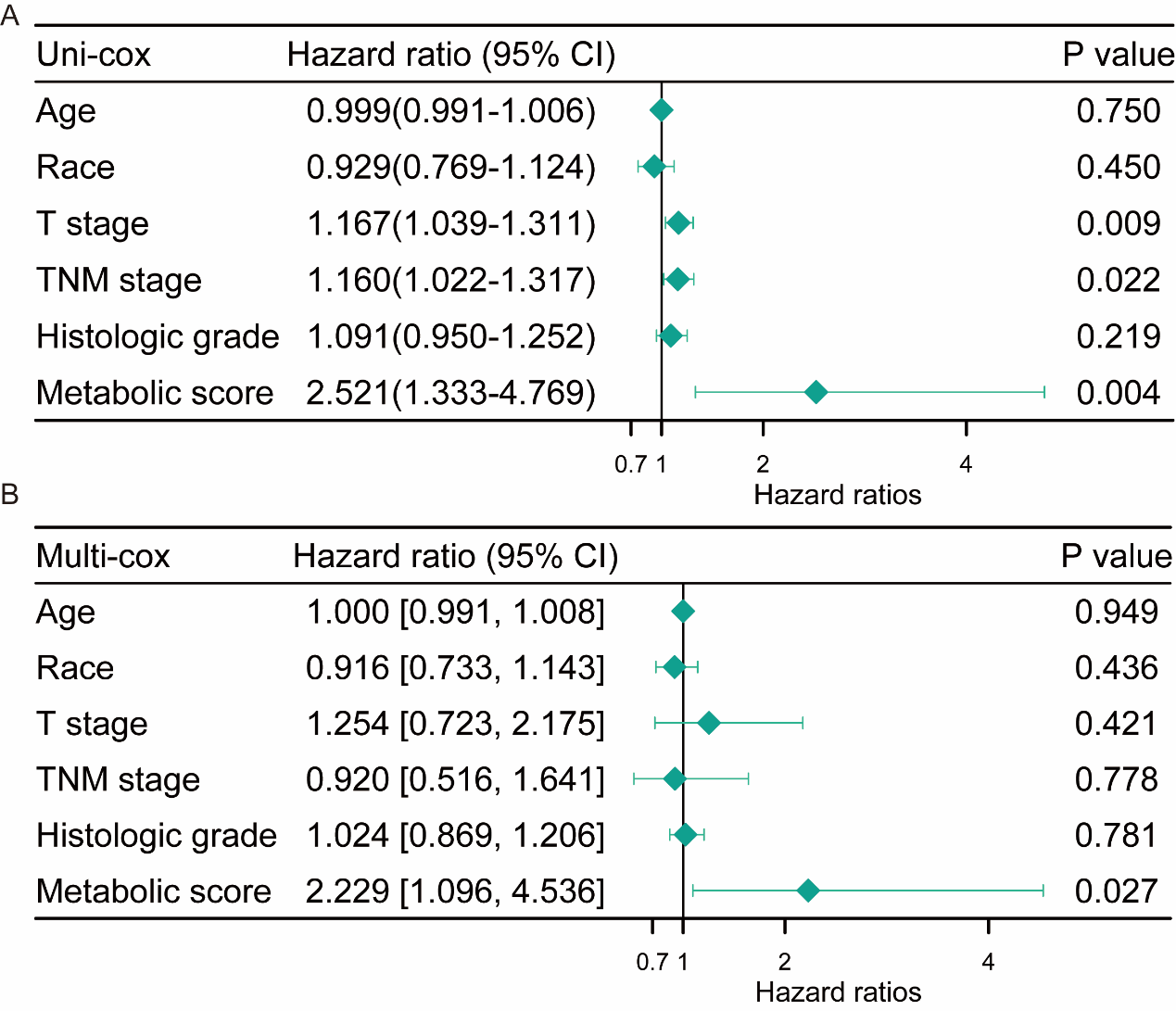
**

**Fig. S12** Univariate COX analysis(A) and Multivariate COX analysis (B) for metabolic scores.


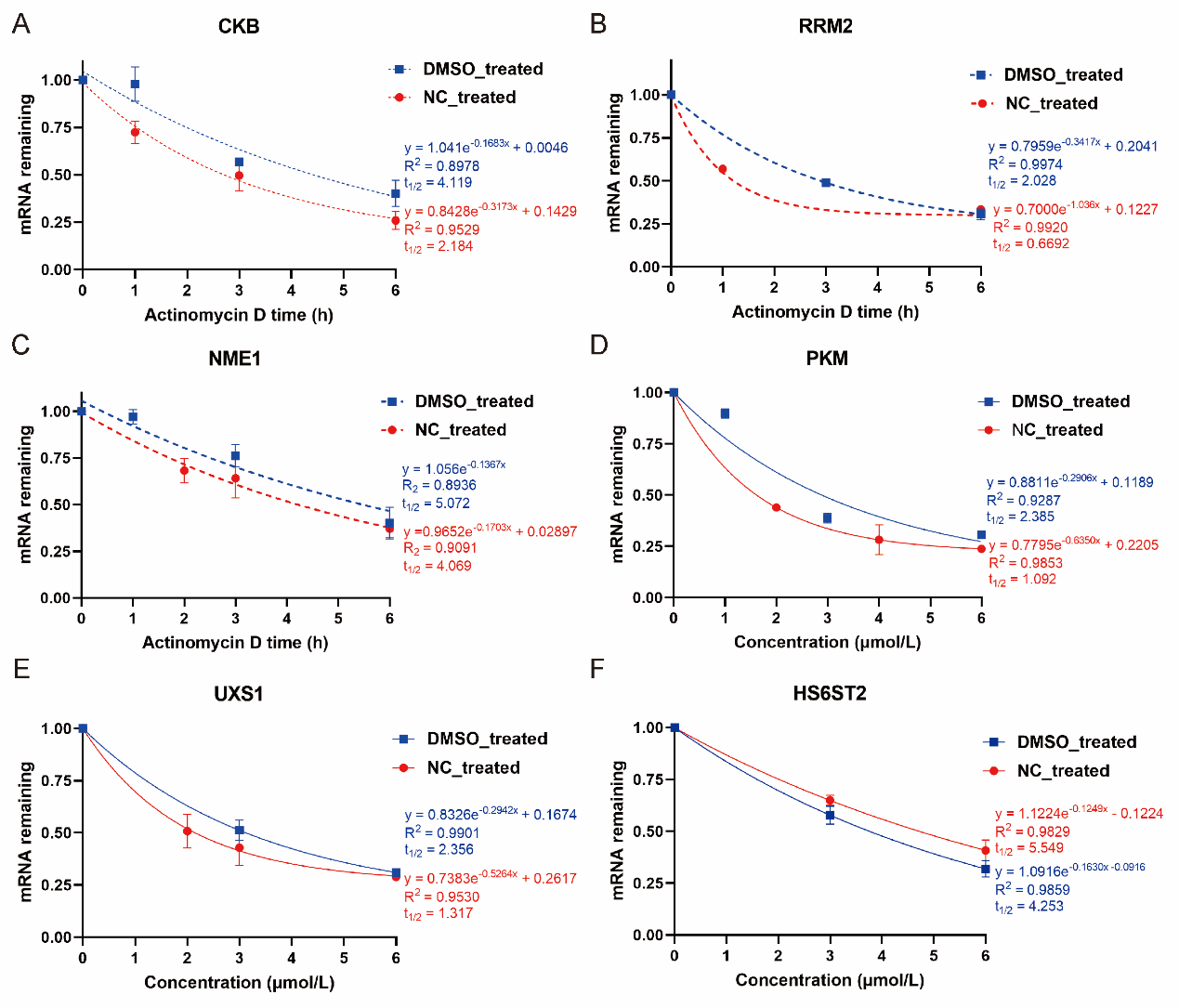


**Fig. S13** RNA stability assay of CKB, RRM2, NME1, PKM, UXS1 and HS6ST2 in Huh7 cells following nitidine chloride treatment. Cells were treated with 5 μg/mL actinomycin D for 0 h, 1 h, 2 h, 3 h, 4 h or 6 hours prior to RNA extraction.
