# Supplementary figures and images for: Nitidine chloride inhibits the progression of hepatocellular carcinoma by suppressing IGF2BP3 and modulates metabolic pathways in an m6A-dependent manner
Source: Mol Med. 2025 Feb 5;31:47. doi: 10.1186/s10020-025-01095-8 (PMC11796242; doi:10.1186/s10020-025-01095-8)

# Repeat 1

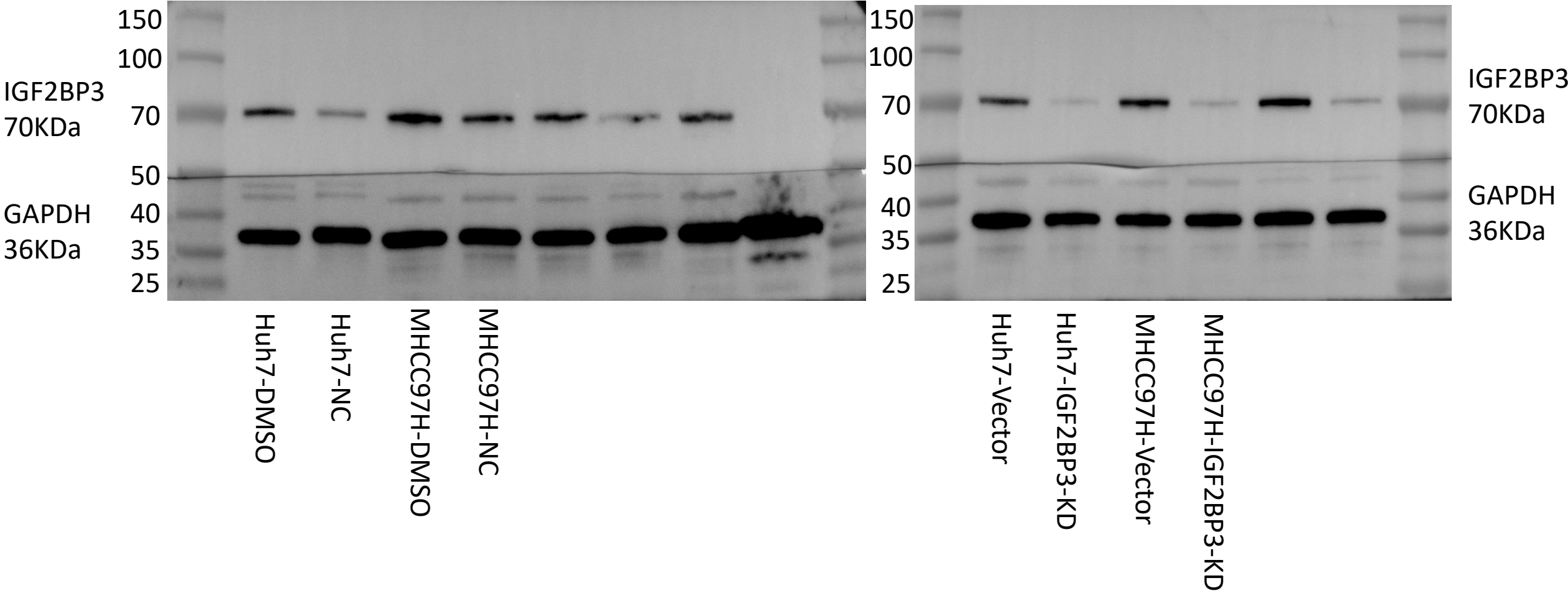

# Repeat 2

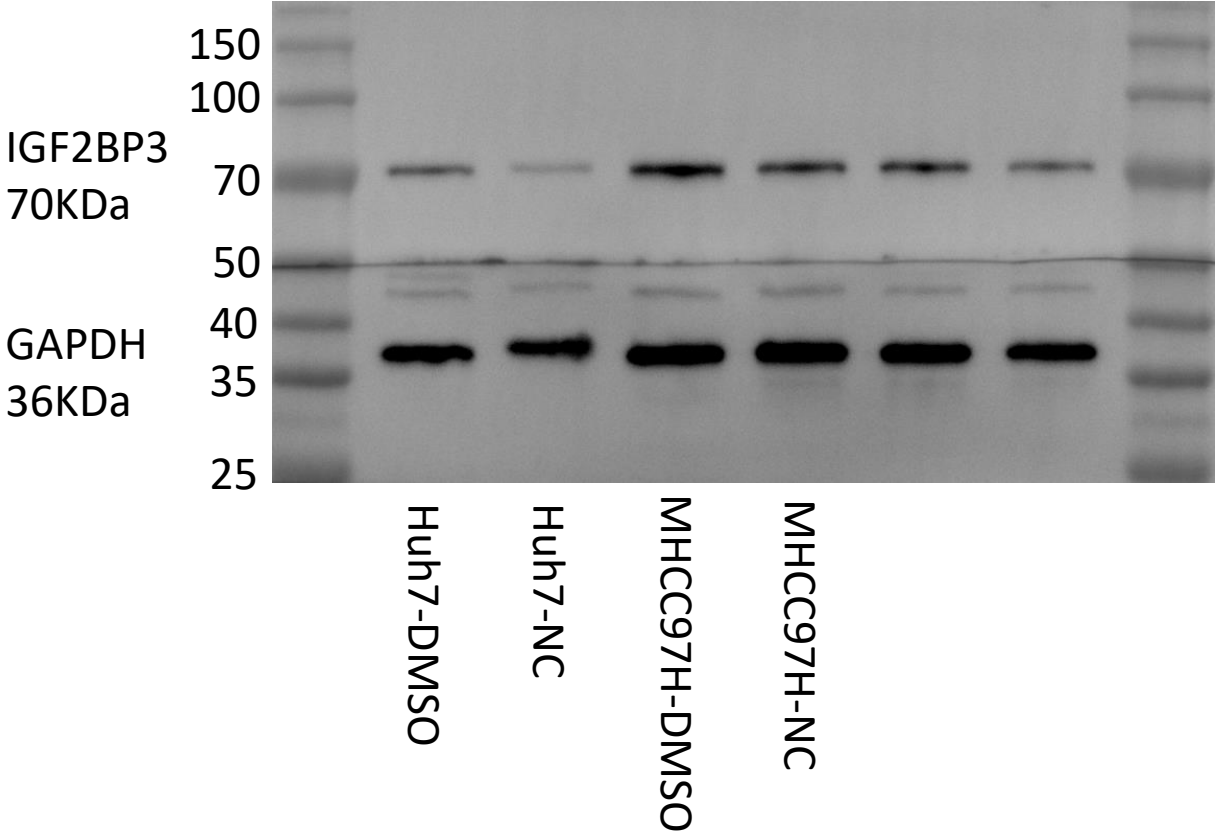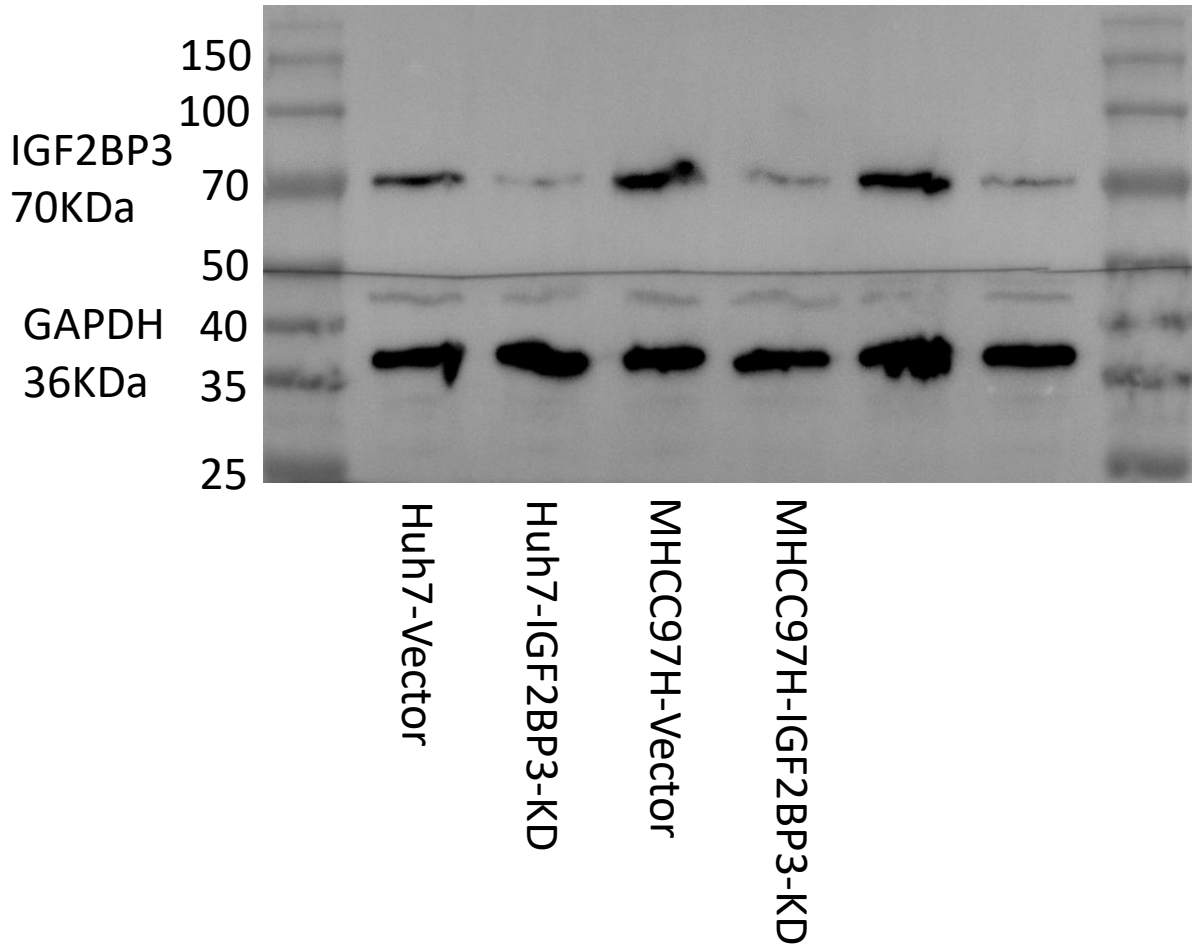

## Repeat 3

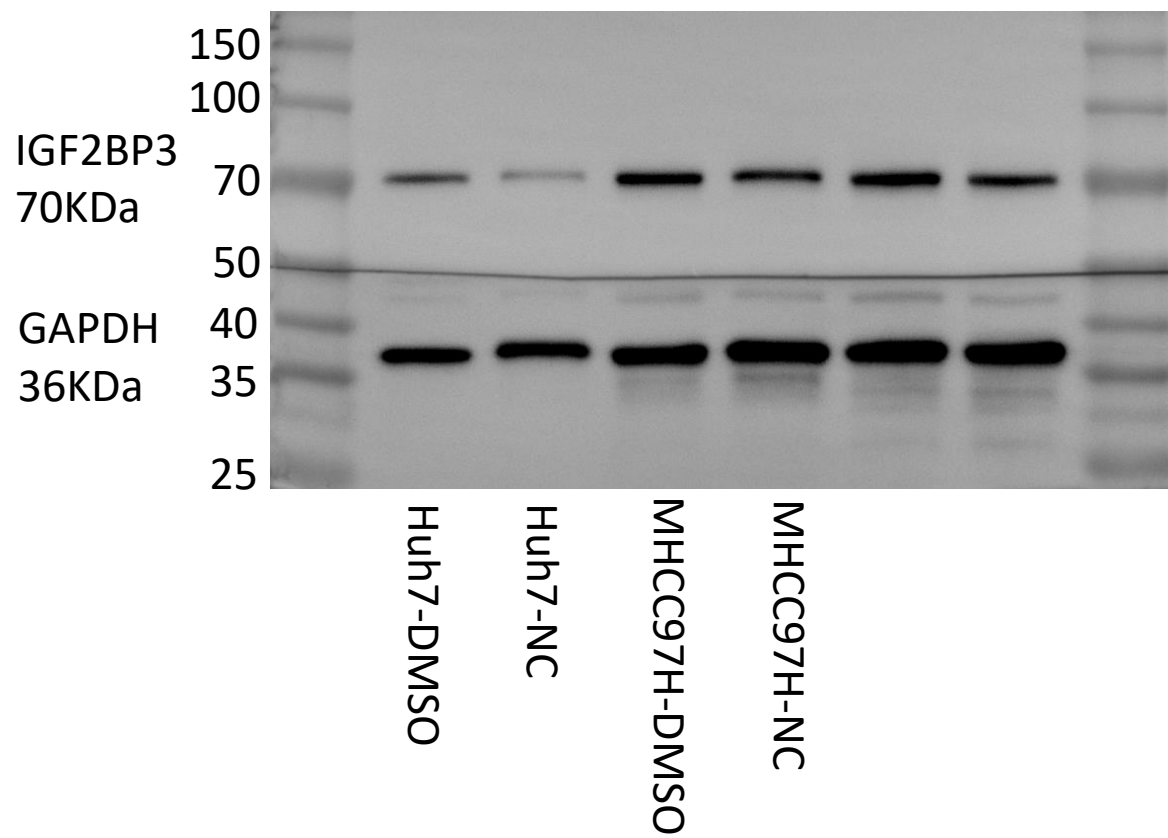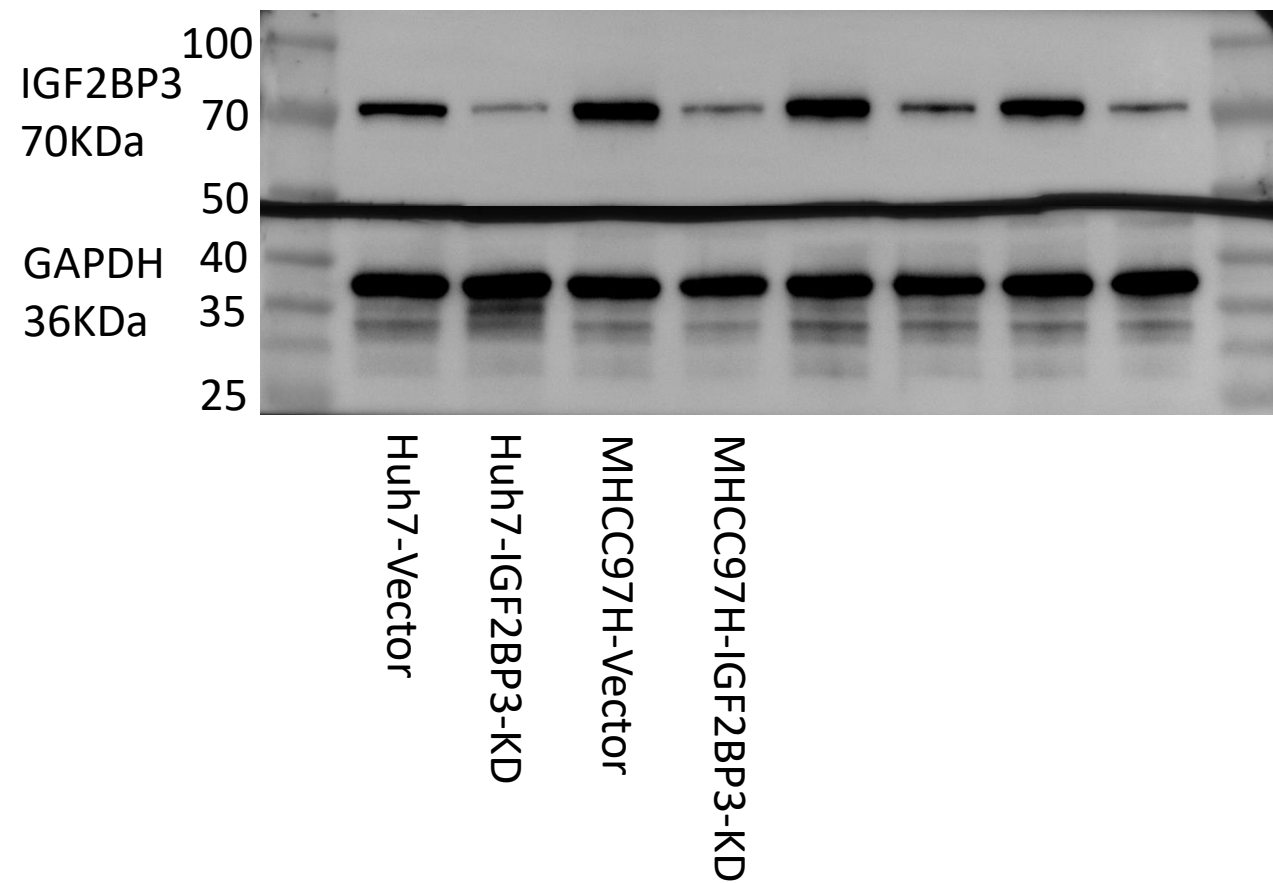

Supplement: Supplementary file 2 — Supplementary Material 2 [file 10020_2025_1095_MOESM2_ESM.pdf]
